# Phylogenetic comparisons of pedestrian locomotion costs: confirmations and new insights

**DOI:** 10.1002/ece3.2267

**Published:** 2016-08-31

**Authors:** Craig R. White, Lesley A. Alton, Taryn S. Crispin, Lewis G. Halsey

**Affiliations:** ^1^ Centre for Geometric BiologySchool of Biological SciencesMonash UniversityMelbourneVictoria3800Australia; ^2^ School of Biological SciencesThe University of QueenslandBrisbaneQueensland4072Australia; ^3^ Department of Life SciencesCentre for Research in EcologyUniversity of RoehamptonHolybourne AvenueLondonSW15 4JDUK

**Keywords:** Locomotion, metabolic rate, oxygen consumption, running, scaling

## Abstract

The energetic costs for animals to locomote on land influence many aspects of their ecology. Size accounts for much of the among‐species variation in terrestrial transport costs, but species of similar body size can still exhibit severalfold differences in energy expenditure. We compiled measurements of the (mass‐specific) minimum cost of pedestrian transport (COT_min_, mL/kg/m) for 201 species – by far the largest sample to date – and used phylogenetically informed comparative analyses to investigate possible eco‐evolutionary differences in COT_min_ between various groupings of those species. We investigated number of legs, ectothermy and endothermy, waddling, and nocturnality specifically in lizards. Thus, our study primarily revisited previous theories about variations in COT_min_ between species, testing them with much more robust analyses. Having accounted for mass, while residual COT_min_ did not differ between bipedal and other species, specifically waddling bipeds were found to have relatively high COT_min_. Furthermore, nocturnal lizards have relatively low COT_min_ although temperature does not appear to affect COT_min_ in ectotherms. Previous studies examining across‐species variation in COT_min_ from a biomechanical perspective show that the differences between waddling birds and nonwaddling species, and between nocturnal lizards and other ecotherms, are likely to be attributable to differences in ground reaction forces, posture, and effective limb length.

## Introduction

Many animals spend a substantial part of their time moving around. For them, locomotion is a fundamental aspect of finding food, escaping from predators, attracting mates, dispersing, and migrating. To move themselves, animals must exert force on their surrounding environment to overcome friction and gravity, and this requires energy to be consumed via cellular work. The energetic cost of locomotion can therefore be considerable (Garland 1983; Speakman and Selman 2003; Rezende et al. 2009; Gefen 2011; Scantlebury et al. 2014; Halsey et al. 2015) and may influence an animal's fitness by constraining the amount of energy it can allocate to growth and reproduction. Consequently, understanding what influences the energetic cost of locomotion in animals has been the subject of much research.

**Figure 1:**
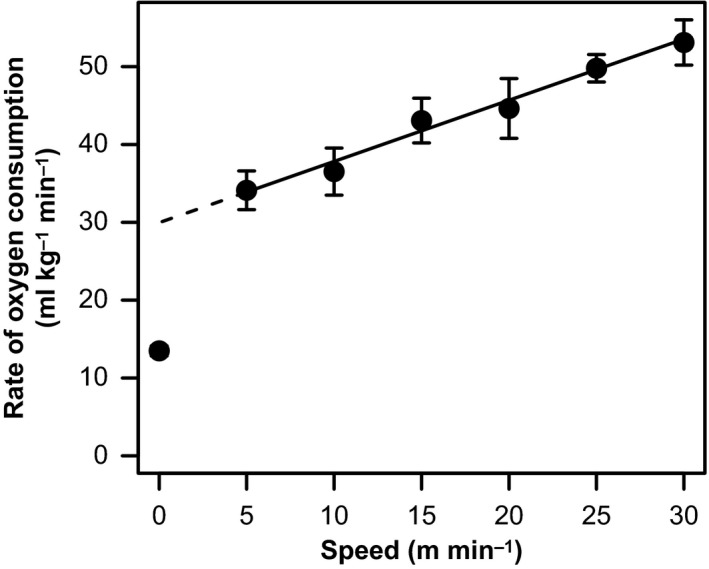
The relationship between rate of oxygen consumption (a proxy for metabolic rate: Lighton and Halsey 2011) and locomotion speed for cormorants *Phalacrocorax carbo* undergoing pedestrian locomotion on a treadmill (data from White et al. 2008a; see also the Supporting information associated with White et al. 2011 for an example of a raw data trace for such an experiment). Filled circles represent mean values of rate of oxygen consumption measured at rest and a range of locomotion speeds and are shown ± SE. The solid line indicates the best fit linear relationship between rate of oxygen consumption and speed during locomotion. The slope of this line represents a speed‐independent minimum cost of transport (COT_min_, mL of O_2_ consumed per kg of body mass per meter traveled), which represents the energy expended over and above the *y*‐intercept of the relationship between metabolic rate and locomotion speed. The dashed line is extrapolated to a speed of 0 m/sec; as is often the case the extrapolated *y*‐intercept falls above the measured resting rate of oxygen consumption (see Halsey 2013 for further discussion of the elevated *y*‐intercept).

An animal's energetic cost of locomotion can be quantified by measuring its metabolic rate (usually as rate of oxygen consumption) while moving at a constant speed, once its cardio‐respiratory physiology has reached steady state. For most, but not all, species, metabolic rate during locomotion is linearly related to speed to at least a good approximation (Taylor et al. 1970, 1982; Schmidt‐Nielsen 1972b), for example, Figure 1. The slope of the linear regression relating metabolic rate and speed represents a speed‐independent minimum cost of transport (COT_min_, mL of O_2_ consumed per kg of body mass per m traveled). COT_min_ estimates the energy expended over and above the *y*‐intercept of the relationship between metabolic rate and speed, where the *y*‐intercept estimates the energetic cost of an animal traveling at a speed of zero: the costs of body maintenance and of holding the body posture associated with movement (see Halsey 2013 for discussion of the *y*‐intercept). Thus, COT_min_ is the theoretical minimum rate of energy expenditure possible by an animal to locomote, that is, if it were able to nullify the costs of other processes not directly related to it moving.

While COT_min_ does not account for all energy costs associated with locomotion, and its calculation assumes a perfect linear relationship between rate of oxygen consumed and locomotion speed, being independent of speed it nonetheless provides an invaluable metric by which to compare movement costs across distantly related and greatly differing animals (Halsey et al. 2016). As is the case for a wide range of physiological traits (Calder 1984; Schmidt‐Nielsen 1984; White and Kearney 2014), a considerable proportion of the among‐species variation in COT_min_ is explained by differences in size between species, with the relationship between COT_min_ and body mass shown to be negative indicating that per unit mass larger animals have a lower COT_min_ (Taylor et al., 1970, Schmidt‐Nielsen 1972a; Full 1989). There are a number of mechanistic investigations discussing the biomechanical and kinematic factors that underlie the relationship between body mass and COT_min_. Kram and Taylor (1990) provide evidence based on five mammal species that COT_min_ is determined primarily by the energy cost to the animal of supporting its body weight and the duration over which the force for doing so is applied to the ground. This manifests as the length of an animal's step during pedestrian locomotion, which is positively related to its body size, being an important determinant of the energy cost of running. Subsequently, Pontzer (2007) showed that the length of the limb as a mechanical strut (effective limb length) is the primary anatomical driver of locomotor costs in terrestrial animals (see also Reilly et al. 2007). Very recently, Pontzer has demonstrated that unifying work‐ and force‐based models centerd on muscle metabolism enhances predictions of COT_min_, not only for running on the flat but also up and down hills, and vertical climbing (Pontzer 2016).

**Figure 2:**
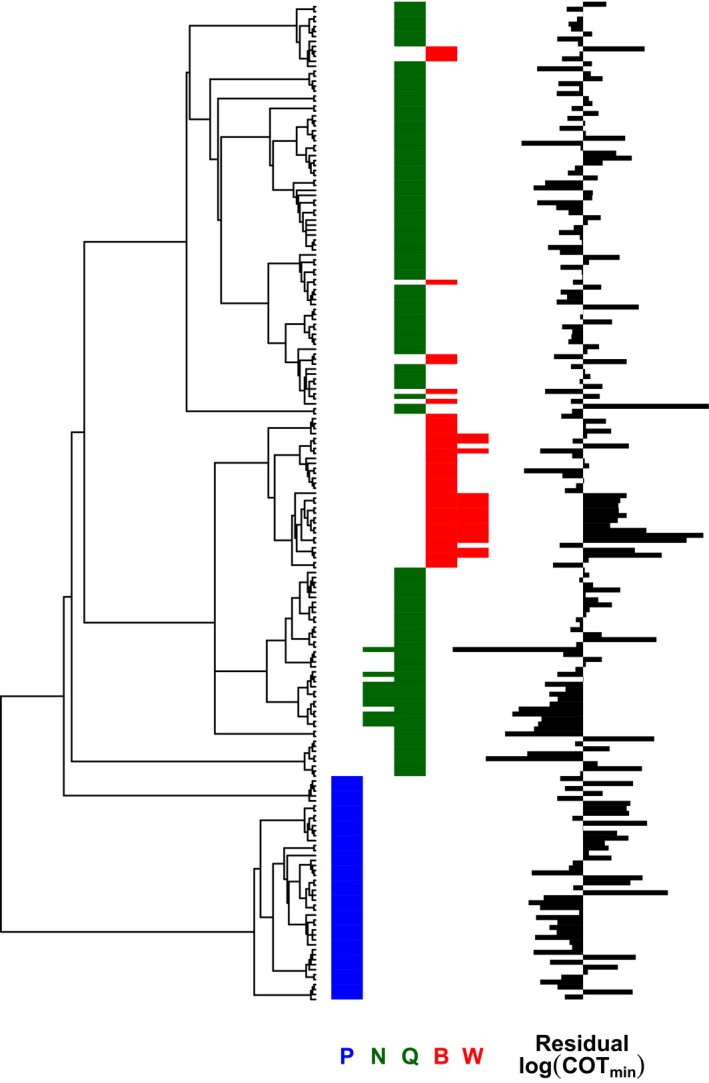
Phylogenetic distribution of polypedalism (P, blue), quadrupedalism (Q, green; specifically nocturnal lizards, N) and bipedalism (B, red; specifically waddling species, W), and mass‐independent residual COT_min_ (black bars). Residual COT_min_ was calculated using the intercept and parameter estimate for body mass from Table 1.

Once the pervasive effect of body mass on COT_min_ is accounted for, however, considerable variation remains. The strength of the logged relationship between COT_min_ and body mass belies the absolute size of many of the residuals; species of similar body mass can have values of COT_min_ that differ by severalfold (Full 1989; Full et al. 1990; Kram 2012). This represents a huge difference in the cost of two similarly sized animals to move a given distance. Indeed, Pontzer's mechanistic model explains 95% of the variance in COT_min_ (Pontzer 2016); yet assessment of data points digitized from Figure 2c in that paper suggests that this impressive relationship still includes up to fivefold mass‐independent differences in absolute COT_min_ for level running alone. For example, the COT_min_ for young lions *Panthera leo* (0.36 mL/kg/m) is calculated to be fourfold higher than that of similarly sized reindeer *Rangifer tarandus* calves (0.09 mL/kg/m; Chassin et al. 1976; Fancy and White 1985; Luick and White 1986). Although Chassin et al. (1976) were unable to account for the high cost of movement in lions, they suggested it may offer a physiological explanation for the reliance of lions on social hunting, which can increase the energy efficiency of obtaining food, in part because larger prey can be killed providing an energy return for multiple individuals in the pride (Williams et al. 2014).

However, it is challenging to infer adaptation from the study of single or small numbers of species because species may differ from one another for a large number of reasons that may or may not be related to the hypothesis of interest (Garland and Adolph 1994). The problems generated by comparisons of small numbers of species are exemplified by considering the COT_min_ of African hunting dogs *Lycaon pictus* (Taylor et al. 1971). Unlike lions, which as mentioned earlier have a relatively high COT_min_, the COT_min_ of African hunting dogs (0.294 mL/kg/m) is very close to that predicted by their body mass (0.290 mL/kg/m; calculated for a mass of 8.75 kg using the parameter estimates in Table 1). This observation suggests that the evolution of social hunting is not always associated with high COT_min_, which calls into question the aforementioned hypothesis put forward by Chassin et al. (1976) that social hunting in lions evolved in part because of their high locomotion costs.

**Table 1:** Parameter estimates for, and importance of, the effects of body mass (M, kg), animals that are bipedal and animals that waddle on log_10_‐transformed minimum cost of transport (mL/kg/m) for ectothermic and endothermic animals, assessed by phylogenetic least squares (maximum likelihood λ = 0 [95% CI: NA, 0.42], *r* ^2^ = 0.85). Importance is calculated as a sum of the Akaike weights over all of the models in which the term appears (see text for details)

Term	Estimate	SE	*t*	Importance
Intercept	−0.28	0.02	−12.85	
Log_10_M	−0.28	0.01	−32.3	1
Waddle	0.31	0.08	4.01	1
Bipedal	−0.002	0.052	−0.03	0.26

Phylogenetically informed comparative analyses offer a strong approach to inferring adaptation by testing for associations among traits across many species while explicitly taking evolutionary history into account (Rezende and Diniz‐Filho 2012). Such analyses seek to reveal the selection pressures that have driven the evolution of interspecies differences and thereby offer an approach that is complementary to biomechanical investigations which reveal the proximate mechanisms by which species achieve these differences. Although the interspecific relationship between body size and the energetics of locomotion has been well studied for more than 40 years (e.g., Taylor et al., 1970; Schmidt‐Nielsen 1972a; Full et al., 1990, Pontzer 2007; White et al. 2008a; Halsey and White 2012), few investigations have examined the scaling of COT_min_ in a phylogenetic context, and those studies including phylogeny have had a narrow taxonomic focus (lizards: Autumn et al. 1997; Hare et al. 2007; mammals: Halsey and White 2012; birds: White et al. 2008a).

In this study, taking advantage of the many relatively recent publications as well as previously compiled data sets to maximize sample size and species diversity, we use phylogenetically informed comparative analyses to test for differences in COT_min_ between various groupings of terrestrial animals that use pedestrian locomotion for movement. Our aim was to identify groups that exhibit different COT_min_ from the typical for terrestrial animals, through examination and quantification of the scaling relationships. We seek to complement proximate mechanistic explanations of COT_min_ variability with ultimate, eco‐evolutionary explanations. We test for differences between groups explored previously (two vs. many legs; Full 1989). We also test for differences in COT_min_ between waddling and nonwaddling species, a comparison that has also been investigated previously. Fedak et al. (1974) and Pinshow et al. (1977) reported higher transport costs in waddling birds; yet, these analyses and others have considered small samples of species and thus provide only limited evidence that waddling is an expensive form of pedestrian locomotion. Furthermore, a recent phylogenetically informed comparison of cormorants *Phalacrocorax carbo*, another species that waddles, with running birds (Galliformes and Struthioniformes) found no significant difference in COT_min_ (White et al. 2008a). We also test the nocturnality hypothesis, which suggests that night‐active lizards are often moving around at low and suboptimal temperatures and will have decreased COT_min_ to overcome the handicap that at lower temperatures, energy is applied to locomotion less efficiently (Autumn et al. 1994, 1997, 1999; Hare et al. 2007). The nocturnality hypothesis is supported by observations of low values of COT_min_ for nocturnal geckos and skinks compared to other species of lizard both closely and distantly related (Autumn et al. 1994, 1997, 1999; Hare et al. 2007); however, an analysis including several nocturnal lizards together has not been undertaken. Furthermore, studies that manipulate temperature and measure the consequences of this for the COT_min_ of ectotherms report that COT_min_ is independent of temperature (e.g., Moberly 1968; Herreid et al. 1981; John‐Alder et al. 1983; Bennett and John‐Alder 1984; Lighton et al. 1993), casting doubt on the nocturnality hypothesis. We investigate the nocturnality hypothesis by testing for an effect in ectotherms of temperature on COT_min_ to determine whether species active at low body temperatures have high COT_min_, as well as testing for differences in COT_min_ between nocturnal lizards and other ectotherms.

## Material and Methods

Data for COT_min_ were compiled from the peer‐reviewed literature and were included only if data for body mass were also available. All data used in this study are available on ResearchGate. COT_min_ was included if the authors of the original study calculated it as the slope of a linear regression relating metabolic rate and locomotion speed or provided data from which this slope could be calculated. The relationship between metabolic rate and locomotion speed is close to linear for most species, but not for all, and thus, we visually assessed the linearity of each data set. At high speeds, metabolic rate is independent of speed for large macropods (e.g., Dawson and Taylor 1973; Baudinette et al. 1992); so, for these species, COT_min_ was calculated as the slope of a linear regression relating metabolic rate and locomotion speed for speeds below that at which metabolic rate becomes independent of speed. Both COT_min_ and body mass were log_10_‐transformed for analysis. In total, we compiled data for 201 species (eight amphibians, five arachnids, 31 birds, four crustaceans, 36 insects, 83 mammals, 34 nonavian reptiles; Fig. 2 and Table in Appendix S1). Sex differences in COT_min_ have been documented for some species (e.g., Browning et al. 2006; Rezende et al. 2006; Lees et al. 2012), but not others (e.g., Shillington and Peterson 2002; Rose et al. 2014), and most studies do not report COT_min_ for males and females separately; we therefore pooled data for males and females. Data were analyzed using phylogenetic generalized least squares (PGLS; Grafen 1989; Martins and Hansen 1997; Garland and Ives 2000) using the “ape” v3.1‐1 (Paradis et al. 2004) and “caper” v0.5.2 (Orme et al. 2013) packages of R v3.0.2 (R Core Team, 2013). The tree used for analysis was constructed using published trees for mammals (Bininda‐Emonds et al. 2007), birds (Jetz et al. 2012), amphibians (Pyron and Wiens 2011), reptiles (Pyron et al. 2013), and insects (Kambhampati 1995; Ward 2007; Misof et al. 2014), supplemented with additional information from tolweb.org (the full tree is provided as the Supporting Information). For birds, a single majority rule consensus tree was constructed from the published posterior distribution of 10,000 trees (Jetz et al. 2012) using “ape” v3.1‐1 (Paradis et al. 2004). Because the branch lengths in the various trees were provided in different units or not provided at all, Grafen's (1989) arbitrary branch length transformation was used (branch lengths set to a length equal to the number of descendant tips minus one). A measure of phylogenetic correlation, λ (Pagel 1999), was estimated by fitting PGLS models with different values of λ and finding the value that maximizes the log likelihood. The degree to which trait evolution deviates from Brownian motion (λ = 1) was determined by modifying the covariance matrix using the maximum likelihood value of λ, which is a multiplier of the off‐diagonal elements of the covariance matrix (i.e., those quantifying the degree of relatedness between species).

For the full data set, the effects of body mass, bipedalism (bipedal or not), and waddling on COT_min_ were examined. We define waddling following (Pinshow et al. 1977) as awkward gaits where the body undergoes large lateral displacements during locomotion and consider waddling species as members of the Anseriformes (ducks, geese, swans, screamers, and the magpie goose), Suliformes (frigate birds, gannets and boobies, cormorants and shags, and darters), Procellariiformes (albatrosses, petrels and shearwaters, storm petrels, and diving petrels), and Sphenisciformes (penguins). For ectotherms, the effects of body temperature and nocturnality (nocturnal lizard, or not) on COT_min_ were also examined. Differences in COT_min_ between nocturnal and non‐nocturnal endotherms were not examined because, in contrast to ectotherms, nocturnal endotherms are not expected to be active at lower body temperatures than non‐nocturnal endotherms.

We estimated the relative importance of size, waddling, and bipedalism (for the full data set) or size, body temperature, and nocturnality (for the ectotherm data set) by fitting models with all possible additive combinations of these predictors and comparing the models within these candidate sets on the basis of the second‐order version of Akaike's information criterion (AIC_c_) as a measure of model fit (Burnham and Anderson 2010). The relative importance weight of each predictor was calculated by summing the Akaike weights (*w*_*i*_, the relative likelihood of the model compared with all others: the likelihood of the model divided by the sum of the likelihoods of all other models) of the models containing the predictor (e.g., the relative importance of body mass was calculated by summing the values of *w*_*i*_ of all models that contained body mass as a predictor; Burnham and Anderson 2010). Relative importance (Σ*w*_*i*_) represents the probability that a given predictor is a component of the best model of the candidate set (Symonds and Moussalli 2011), but should not be interpreted as a threshold metric used to separate weak, moderate, or strong support for predictors because Σ*w*_*i*_ can take a wide range of values even when predictor variables are unrelated to the response (Galipaud et al. 2014). We therefore interpret Σ*w*_*i*_ conservatively, concluding that predictors with Σ*w*_*i*_ = 1 have an influence on COT_min_, and interpreting predictors with Σ*w*_*i*_ < 1 based on the magnitude of their estimated biological effect on COT_min_.

## Results

**Figure 3:**
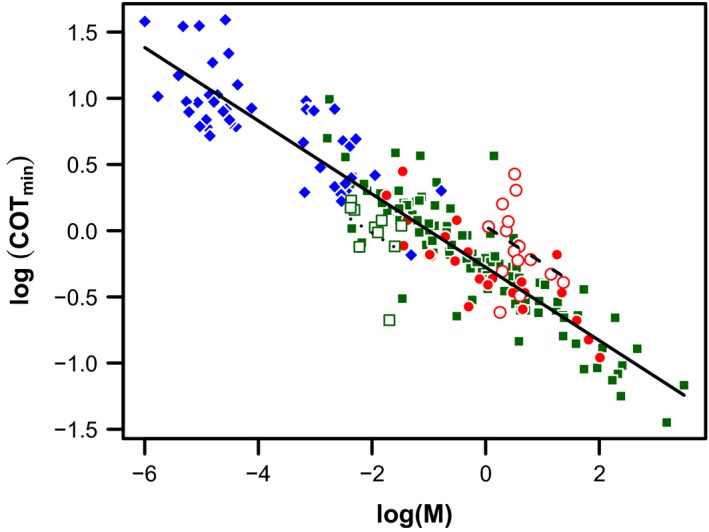
Scaling of log_10_‐transformed cost of transport (COT_min_, mL/kg/m) with log_10_‐transformed body mass (M, kg) in bipedal (red circles), quadrupedal (green squares), and polypedal (blue diamonds) species; unfilled red symbols represent waddling species and unfilled green symbols represent nocturnal lizards. The dashed and solid lines represent the relationship between COT_min_ and mass for waddling and all remaining species, respectively (Table 1); the dotted line represents the relationship for nocturnal lizards (Table 2).

With data for all species included, there was an effect of body mass on COT_min_ (Σ*w*_*i*_ = 1.00, Fig. 3, Table 1), and a residual difference between waddling bipedal species and all other species (Σ*w*_*i*_ = 1; waddling species have an COT_min_ that is about twofold higher than other species, Fig. 3, Table 1). However, there was essentially no difference between bipedal species in general and other species (Σ*w*_*i*_ = 0.26; bipedal species have a mean COT_min_ that differs from nonbipedal species of similar mass by <1%; Fig. 3, Table 1).

**Figure 4:**
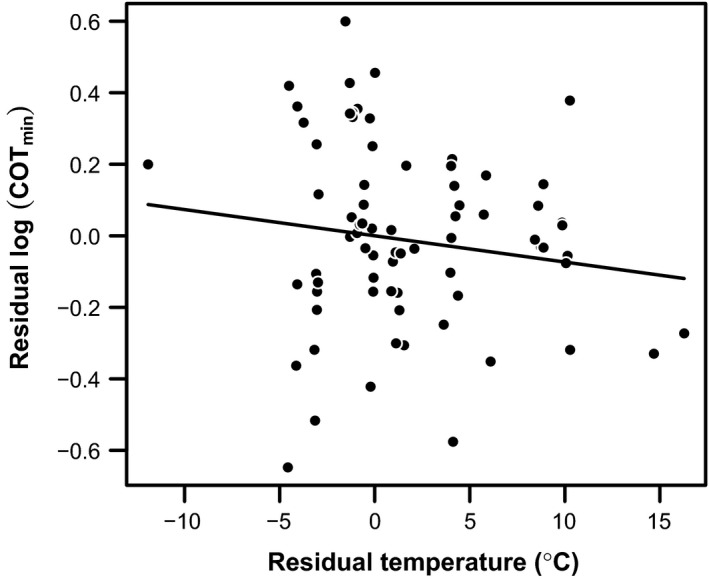
The among‐species relationship between body temperature (°C) and minimum cost of transport (COT_min_) for ectotherms. Temperature and COT_min_ are presented as residuals to account for the influence of other predictors on COT_min_ (Table 2). The solid line represents the parameter estimate for the effect of temperature from Table 2, plotted through the origin (0,0).

When only data for ectotherms were considered, there was an effect of body mass (Σ*w*_*i*_ = 1.00, Table 2) and a difference between nocturnal lizards and other ectotherms (Σ*w*_*i*_ = 0.91; nocturnal lizards have a mean COT_min_ that is 47% of that for other ectotherms of similar mass: Fig. 3, Table 2). There was only a small effect of temperature on COT_min_ (Σ*w*_*i*_ = 0.41; mean COT_min_ of ectotherms decreases by ~2%/°C: Table 2, Fig. 4).

**Table 2:** Parameter estimates and importance for the effects of body mass (M, kg), body temperature (°C), and nocturnality on log_10_‐transformed minimum cost of transport (mL/kg/m) for ectothermic animals, assessed by phylogenetic generalized least squares (maximum likelihood λ = 0 [95% CI: NA, 0.67], *r* ^2^ = 0.79). Importance is calculated as a sum of the Akaike weights over all of the models in which the term appears (see text for details)

Term	Estimate	SE	*t*	Importance
Intercept	0.07	0.17	0.40	
Log_10_M	−0.27	0.02	−14.4	1
Nocturnality	−0.32	0.09	−3.52	0.91
Temperature	−0.007	0.006	−1.23	0.41

## Discussion

As shown in the present study (Fig. 3) and others (e.g., Schmidt‐Nielsen 1972a; Full et al., 1990), COT_min_ for leg‐based movement on land decreases with increasing size. The negative relationship between COT_min_ and size across species, which has an allometric scaling exponent of −0.28, probably arises mainly because small animals have higher stride frequencies than large ones (Heglund and Taylor 1988; Gatesy and Biewener 1991) and therefore have less time available during each stride to generate force against the ground (Kram and Taylor 1990). High rates of force generation require the recruitment of faster, less economical muscle fibers (Huxley 1974; Rall 1985; Kram and Taylor 1990; Griffin and Kram 2000) and perhaps also the generation of force more quickly than is optimal for those fibers that are activated (Bárány 1967), thereby increasing COT_min_.

However, animals of a similar size present in our data set exhibit considerable variation in their energy economy (Figs. 2, 3), routinely representing sevenfolds of difference. Some of these differences in COT_min_ may be explained by certain species running relatively poorly on a treadmill, the use of juvenile animals, measurement error, and the assumption of perfect linearity in the derivation of COT_min_. However, much of the variation is likely to be genuine, and these among‐species differences have been proposed as important in the evolution of a range of ecological patterns. Our study confirms and progresses understanding of which eco‐evolutionary traits independent of body mass are associated with COT_min_. Our phylogenetically informed analysis across 201 terrestrial species confirmed the lack of evidence for a difference in COT_min_ between species with two legs and species with more, which fits with the present biomechanical theories that locomotor costs are driven by supporting body weight (Kram and Taylor 1990) and moderated by step, limb, and limb muscle length (Kram and Taylor 1990; Roberts et al. 1998a,b; Pontzer 2007). Our analyses also adds considerable weight to the relatively limited previous evidence that waddling bipeds have a higher COT_min_ than do other animals (Fig. 3, Table 1), indicating that it is approximately double. It has previously been suggested that the ultimate explanation for this greater COT_min_ may be that waddling species such as penguins, ducks, geese, and cormorants have evolved short legs in association with aquatic specialization (White et al. 2008a). Thus, from a proximate biomechanical standpoint, the cost of generating force probably also explains the generally high COT_min_ in waddling bipeds, because their short legs necessitate high rates of force generation during short strides (Griffin and Kram 2000; see also Pontzer 2007). An interesting avenue for further work would therefore be to determine whether additional residual variation in COT_min_ can be explained by among‐species differences in the time course of force generation. Furthermore, within ectotherms, we found support for the nocturnality hypothesis (Autumn et al. 1994, 1997, 1999; Hare et al. 2007), suggesting that poikilothermic species of lizard which forage at night are substantially more energetically economic than other ectotherm species (our analyses suggest their COT_min_ is about half), although there was little support for the related hypothesis that COT_min_ changes with body temperature (Table 2, Fig. 4).

The lack of a relationship between COT_min_ and temperature in ectotherms is consistent with the results of all single‐species studies that we are aware of (e.g., Moberly 1968; Herreid et al. 1981; John‐Alder et al. 1983; Bennett and John‐Alder 1984; Lighton et al. 1993). Furthermore, the parameter estimate for temperature corresponding to a 20% decrease in COT_min_ per 10°C of temperature increase has low precision and is small compared to the 1.5‐ to threefold changes in physiological rates that are typically caused by acute 10°C changes in temperature (Seebacher et al. 2015). The finding that COT_min_ is largely independent of body temperature (Table 2) despite the profound effect of temperature on rates of physiological processes (Dell et al. 2011) again supports the hypothesis that COT_min_ is influenced primarily by the cost of generating force to support the body against gravity (Kram and Taylor 1990). Furthermore, this finding goes against the nocturnality hypothesis, which suggests that nocturnal lizards, which evolved from diurnal species active at high body temperatures, are often active at low and suboptimal temperatures and therefore have decreased COT_min_ to overcome the reduced performance observed at low temperatures (Autumn et al. 1994, 1997, 1999; Hare et al. 2007). Despite this, the probability that the nocturnality predictor is present in the best model in the candidate set of our analysis is 0.91 and nocturnal lizards have a mean COT_min_ that is 47% of the COT_min_ of other ectotherms of similar mass (Fig. 3, Table 2). This is fairly clear evidence that nocturnal lizards have a low COT_min_. Intriguingly, though, the low COT_min_ is unlikely to arise as a consequence of selection acting to ameliorate temperature‐mediated changes in COT_min_ directly, as proposed by the nocturnality hypothesis, because COT_min_ is independent of body temperature. Instead, the changes in COT_min_ must arise as a correlated response to selection on other traits. Maximum aerobic metabolic rate is typically thermally dependent at low temperatures in reptiles (e.g., Bennett 1982; Autumn et al. 1994; White et al. 2008b); so, all else being equal, decreases in temperature will therefore lead to reductions in the maximum speed that can be sustained aerobically. Selection to reduce COT_min_ or increase aerobic capacity should overcome this limitation of low‐temperature activity (Autumn et al. 1994). A future avenue for research should be to investigate the physiological and/or biomechanical underpinnings that enable this energy economy in nocturnal lizards; again, we suggest prioritizing investigation of limb length and the time course of force generation.

## Conflict of Interest

None declared.

## Supporting information


**Appendix S1.** Full data set and phylogenetic information.
