# Supplementary material for: Phylogenetic comparisons of pedestrian locomotion costs: confirmations and new insights
Source: Ecol Evol. 2016 Aug 31;6(18):6712–20. doi: 10.1002/ece3.2267 (PMC5058540; doi:10.1002/ece3.2267)
Supplement: Supplementary file 1 — Appendix S1. Full data set and phylogenetic information. [file ECE3-6-6712-s001.docx]

**Appendix - Table and Figure**. Data used in the analyses of the relationship between minimum cost of transport, calculated as the slope (ml kg^-1^ m^-1^) of the linear relationship between mass-specific rate of oxygen consumption (ml min^-1^) and speed (m min^-1^), and body mass (kg). Columns indicate pedalism (Ped), bipedalism (Bi.), ednothermy (Endo), waddling (Wad), Nocturnality (Noc), temperature (T, °C), mass (kg), slope (NCOT, the slope of the relationship between rate of oxygen consumption and speed, ml kg^-1^ m^-1^) and intercept (Int, the intercept of the relationship between rate of oxygen consumption and speed (ml g^-1^ h^-1^). The figure represents the phylogenetic tree applied to the data in the present study.

| **Species** | **Group** | **Subgroup** | **Ped** | **Bi** | **Endo** | **Wad** | **Noc** | **T** | **Mass** | **Slope** | **Int.** | **Reference** |
| --- | --- | --- | --- | --- | --- | --- | --- | --- | --- | --- | --- | --- |
| *Coenobita compressus* | Crustacea | Crustacea | Poly | 0 | 0 | 0 | 0 | 22 | 0.0035 | 2.000 | 0.120 | ([Herreid and Full, 1986](#_ENREF_82)) |
| *Cardisoma guanhumi* | Crustacea | Crustacea | Poly | 0 | 0 | 0 | 0 | 25 | 0.1655 | 2.000 | NA | ([Herreid et al., 1979](#_ENREF_84)) |
| *Ocypode gaudichaudii* | Crustacea | Crustacea | Poly | 0 | 0 | 0 | 0 | 22 | 0.00278 | 1.900 | 0.380 | ([Full and Herreid, 1983](#_ENREF_59)) |
| *Ocypode quadrata* | Crustacea | Crustacea | Poly | 0 | 0 | 0 | 0 | NA | 0.0489 | 0.655 | NA | ([Full, 1987](#_ENREF_56)) |
| *Anthia fabricii* | Insecta | Insecta | Poly | 0 | 0 | 0 | 0 | 22 | 0.0022 | 2.150 | 0.550 | ([Lighton, 1985](#_ENREF_101)) |
| *Calosoma affine* | Insecta | Insecta | Poly | 0 | 0 | 0 | 0 | 23.5 | 0.00062 | 4.636 | 0.269 | ([Full et al., 1990b](#_ENREF_61)) |
| *Pachysoma hippocrates* | Insecta | Insecta | Poly | 0 | 0 | 0 | 0 | 22 | 0.0031 | 4.760 | 0.110 | ([Lighton, 1985](#_ENREF_101)) |
| *Psammodes striatus* | Insecta | Insecta | Poly | 0 | 0 | 0 | 0 | 22 | 0.0029 | 1.670 | 0.230 | ([Lighton, 1985](#_ENREF_101)) |
| *Onymacris plana* | Insecta | Insecta | Poly | 0 | 0 | 0 | 0 | 35 | 0.000691 | 9.542 | 0.466 | ([Bartholomew et al., 1985](#_ENREF_8)) |
| *Physadesima globosa* | Insecta | Insecta | Poly | 0 | 0 | 0 | 0 | 35 | 0.000652 | 1.947 | 1.757 | ([Bartholomew et al., 1985](#_ENREF_8)) |
| *Physosterna cribripes* | Insecta | Insecta | Poly | 0 | 0 | 0 | 0 | 35 | 0.001226 | 2.998 | 0.520 | ([Bartholomew et al., 1985](#_ENREF_8)) |
| *Pachycondyla berthoudi* | Insecta | Insecta | Poly | 0 | 0 | 0 | 0 | 25 | 0.000027 | 8.262 | 0.215 | ([Duncan, 1999](#_ENREF_37)) |
| *Leptogenys nitida* | Insecta | Insecta | Poly | 0 | 0 | 0 | 0 | 25 | 0.0000017 | 10.329 | 0.592 | ([Duncan and Crewe, 1993](#_ENREF_38)) |
| *Leptogenys schwabi* | Insecta | Insecta | Poly | 0 | 0 | 0 | 0 | 25 | 0.0000085 | 9.298 | 0.520 | ([Duncan and Crewe, 1993](#_ENREF_38)) |
| *Leptogenys attenuata* | Insecta | Insecta | Poly | 0 | 0 | 0 | 0 | 25 | 0.0000054 | 9.412 | 0.717 | ([Duncan and Crewe, 1993](#_ENREF_38)) |
| *Megaponera foetens majors* | Insecta | Insecta | Poly | 0 | 0 | 0 | 0 | 25 | 0.0000404 | 6.111 | 0.592 | ([Duncan, 1995](#_ENREF_36)) |
| *Megaponera foetens minors* | Insecta | Insecta | Poly | 0 | 0 | 0 | 0 | 25 | 0.0000124 | 6.051 | 0.968 | ([Duncan, 1995](#_ENREF_36)) |
| *Paraponera clavata* | Insecta | Insecta | Poly | 0 | 0 | 0 | 0 | 28 | 0.000019 | 10.603 | 0.018 | ([Fewell et al., 1996](#_ENREF_48)) |
| *Myrmecocystus mendax* | Insecta | Insecta | Poly | 0 | 0 | 0 | 0 | 40 | 0.000006 | 7.878 | 1.004 | ([Duncan and Lighton, 1994](#_ENREF_39)) |
| *Myrmecocystus mexicanus* | Insecta | Insecta | Poly | 0 | 0 | 0 | 0 | 30 | 0.000014 | 5.204 | 0.305 | ([Duncan and Lighton, 1994](#_ENREF_39)) |
| *Camponotus sp* | Insecta | Insecta | Poly | 0 | 0 | 0 | 0 | 27.5 | 0.000012 | 6.885 | 0.666 | ([Lipp et al., 2005](#_ENREF_108)) |
| *Camponotus herculaneus* | Insecta | Insecta | Poly | 0 | 0 | 0 | 0 | 22.5 | 0.00002625 | 39.104 | NA | ([Jensen and Holm-Jensen, 1980](#_ENREF_88)) |
| *Camponotus rufipes* | Insecta | Insecta | Poly | 0 | 0 | 0 | 0 | 25 | 0.0000137 | 10.573 | NA | ([Schilman and Roces, 2005](#_ENREF_140)) |
| *Formica fusa* | Insecta | Insecta | Poly | 0 | 0 | 0 | 0 | 22.5 | 0.0000047 | 34.905 | NA | ([Jensen and Holm-Jensen, 1980](#_ENREF_88)) |
| *Formica rufa* | Insecta | Insecta | Poly | 0 | 0 | 0 | 0 | 22.5 | 0.0000091 | 35.214 | NA | ([Jensen and Holm-Jensen, 1980](#_ENREF_88)) |
| *Pogonomyrmex maricopa* | Insecta | Insecta | Poly | 0 | 0 | 0 | 0 | 38.5 | 0.0000093 | 6.125 | NA | ([Weier et al., 1995](#_ENREF_164)) |
| *Pogonomyrmex rugosus* | Insecta | Insecta | Poly | 0 | 0 | 0 | 0 | 34 | 0.0000165 | 9.373 | 0.896 | ([Lighton and Feener, 1989](#_ENREF_104)) |
| *Messor capensis* | Insecta | Insecta | Poly | 0 | 0 | 0 | 0 | 25 | 0.0000039 | 14.844 | 0.553 | ([Clusella-Trullas et al., 2010](#_ENREF_31)) |
| *Atta colombica* | Insecta | Insecta | Poly | 0 | 0 | 0 | 0 | 28 | 0.0000156 | 18.600 | NA | ([Lighton et al., 1987](#_ENREF_102)) |
| *Dasymutilla gloriosa* | Insecta | Insecta | Poly | 0 | 0 | 0 | 0 | 30 | 0.000076 | 8.411 | 0.592 | ([Duncan and Lighton, 1997](#_ENREF_40)) |
| *Drosophila melanogaster* | Insecta | Insecta | Poly | 0 | 0 | 0 | 0 | 25 | 0.000001 | 38.000 | NA | ([Berrigan and Patridge, 1997](#_ENREF_22)) |
| *Protophormia terraenovae* | Insecta | Insecta | Poly | 0 | 0 | 0 | 0 | 30 | 0.0000431 | 12.649 | 2.116 | ([Berrigan and Lighton, 1994](#_ENREF_21)) |
| *Periplaneta americana* | Insecta | Insecta | Poly | 0 | 0 | 0 | 0 | 24.5 | 0.000755 | 8.300 | NA | ([Herreid and Full, 1984](#_ENREF_81), [Full et al., 1990b](#_ENREF_61)) |
| *Blaberus discoidalis* | Insecta | Insecta | Poly | 0 | 0 | 0 | 0 | 25 | 0.004415 | 4.35 | 0.71 | ([Full and Tullis, 1990](#_ENREF_60), [Herreid and Full, 1984](#_ENREF_81)) |
| *Blaberus giganteus* | Insecta | Insecta | Poly | 0 | 0 | 0 | 0 | 26 | 0.00433 | 2.500 | 0.029 | ([Bartholomew and Lighton, 1985](#_ENREF_7)) |
| *Eublaberus posticus* | Insecta | Insecta | Poly | 0 | 0 | 0 | 0 | 25 | 0.0022 | 8.290 | 0.050 | ([Herreid and Full, 1984](#_ENREF_81)) |
| *Gromphadorhina chopardi* | Insecta | Insecta | Poly | 0 | 0 | 0 | 0 | 25 | 0.0034 | 2.270 | 0.280 | ([Herreid and Full, 1984](#_ENREF_81)) |
| *Gromphadorhina portentosa* | Insecta | Insecta | Poly | 0 | 0 | 0 | 0 | 24 | 0.0052 | 4.920 | 0.450 | ([Herreid et al., 1981](#_ENREF_83)) |
| *Gryllus bimaculatus* | Insecta | Insecta | Poly | 0 | 0 | 0 | 0 | 21 | 0.0007 | 8.248 | 0.207 | ([Fleming and Bateman, 2007](#_ENREF_51)) |
| *Teleogryllus commodus* | Insecta | Insecta | Poly | 0 | 0 | 0 | 0 | 23.5 | 0.00095 | 8.038 | 1.094 | ([Full et al., 1990b](#_ENREF_61)) |
| *Dinothrombium magnificum* | Arachnida | Arachnida | Poly | 0 | 0 | 0 | 0 | 24 | 0.000032 | 6.499 | 0.197 | ([Lighton and Duncan, 1995](#_ENREF_103)) |
| *Aphonopelma anax* | Arachnida | Arachnida | Poly | 0 | 0 | 0 | 0 | NA | 0.011315 | 2.620 | 0.038 | ([Shillington and Peterson, 2002](#_ENREF_147)) |
| *Myrmecotypus rettenmyeri* | Arachnida | Arachnida | Poly | 0 | 0 | 0 | 0 | 28 | 0.000024 | 7.948 | 2.008 | ([Lighton and Gillespie, 1989](#_ENREF_105)) |
| *Marpissa muscosa* | Arachnida | Arachnida | Poly | 0 | 0 | 0 | 0 | 20 | 0.00003 | 21.798 | 0.735 | ([Schmitz, 2005](#_ENREF_144)) |
| *Pardosa lugubris* | Arachnida | Arachnida | Poly | 0 | 0 | 0 | 0 | 20 | 0.000031 | 6.873 | 1.004 | ([Schmitz, 2005](#_ENREF_144)) |
| *Bufo woodhousii* | Amphibia | Amphibia | Quad | 0 | 0 | 0 | 0 | 21 | 0.0258 | 3.870 | 0.220 | ([Walton and Anderson, 1988](#_ENREF_162)) |
| *Rhinella marina* | Amphibia | Amphibia | Quad | 0 | 0 | 0 | 0 | 23 | 0.21 | 1.080 | NA | ([Baudinette et al., 2000](#_ENREF_13)) |
| *Bufo boreas* | Amphibia | Amphibia | Quad | 0 | 0 | 0 | 0 | 24.5 | 0.1006 | 1.006 | 0.280 | ([Walton et al., 1994](#_ENREF_161)) |
| *Ambystoma tigrinum* | Amphibia | Amphibia | Quad | 0 | 0 | 0 | 0 | 21 | 0.0342 | 0.307 | 0.251 | ([Full et al., 1988](#_ENREF_58)) |
| *Ambystoma laterale* | Amphibia | Amphibia | Quad | 0 | 0 | 0 | 0 | 21 | 0.0044 | 1.039 | 0.204 | ([Full et al., 1988](#_ENREF_58)) |
| *Desmognathus ochrophaeus* | Amphibia | Amphibia | Quad | 0 | 0 | 0 | 0 | NA | 0.00178 | 9.828 | NA | ([Feder, 1986](#_ENREF_46), [Full et al., 1988](#_ENREF_58)) |
| *Plethodon jordani* | Amphibia | Amphibia | Quad | 0 | 0 | 0 | 0 | 27.2 | 0.0041 | 2.250 | 0.100 | ([Full, 1986](#_ENREF_55)) |
| *Bolitoglossa subpalmata* | Amphibia | Amphibia | Quad | 0 | 0 | 0 | 0 | 13 | 0.00163 | 4.993 | 0.109 | ([Feder, 1987](#_ENREF_47)) |
| *Terrapene ornata* | NonAvianReptile | NonAvianReptile | Quad | 0 | 0 | 0 | 0 | 22.9 | 0.31 | 0.226 | 0.112 | ([Zani and Kram, 2008](#_ENREF_177)) |
| *Emydura macquarii* | NonAvianReptile | NonAvianReptile | Quad | 0 | 0 | 0 | 0 | 23 | 0.58 | 0.300 | NA | ([Baudinette et al., 2000](#_ENREF_13)) |
| *Struthio camelus* | Aves | OtherBirds | Bi | 1 | 1 | 0 | 0 | NA | 103 | 0.110 | 0.324 | ([Fedak and Seeherman, 1979](#_ENREF_45)) |
| *Rhea americana* | Aves | OtherBirds | Bi | 1 | 1 | 0 | 0 | NA | 22 | 0.340 | 0.390 | ([Taylor et al., 1971a](#_ENREF_153), [Fedak et al., 1974](#_ENREF_44)) |
| *Nothoprocta pentlandii* | Aves | OtherBirds | Bi | 1 | 1 | 0 | 0 | NA | 0.31 | 1.200 | 0.697 | ([Fedak et al., 1974](#_ENREF_44)) |
| *Dromaius novaehollandiae* | Aves | OtherBirds | Bi | 1 | 1 | 0 | 0 | NA | 40.1 | 0.210 | NA | ([Roberts et al., 1998c](#_ENREF_137)) |
| *Cairina moschata* | Aves | OtherBirds | Bi | 1 | 1 | 0 | 0 | NA | 3.8 | 0.792 | 0.698 | ([Halsey et al., 2009](#_ENREF_76)) and Halsey et al. unpub. |
| *Somateria mollissima* | Aves | OtherBirds | Bi | 1 | 1 | 1 | 0 | NA | 1.79 | 0.242 | 1.728 | ([Hawkins et al., 2000](#_ENREF_79), [Zeffer et al., 2003](#_ENREF_178)) |
| *Anser anser* | Aves | OtherBirds | Bi | 1 | 1 | 1 | 0 | NA | 4.015 | 0.322 | 0.766 | ([Halsey et al., 2009](#_ENREF_76)) and Halsey et al. unpub. |
| *Branta leucopsis* | Aves | OtherBirds | Bi | 1 | 1 | 1 | 0 | NA | 1.963125 | 0.493 | 0.796 | ([Nolet et al., 1992](#_ENREF_116)) |
| *Numida meleagris* | Aves | OtherBirds | Bi | 1 | 1 | 0 | 0 | NA | 1.3235 | 0.435 | 1.516 | ([Fedak et al., 1974](#_ENREF_44), [Ellerby et al., 2003](#_ENREF_41)) |
| *Colinus virginianus* | Aves | OtherBirds | Bi | 1 | 1 | 0 | 0 | NA | 0.194 | 0.900 | 1.400 | ([Fedak et al., 1974](#_ENREF_44)) |
| *Gallus gallus* | Aves | OtherBirds | Bi | 1 | 1 | 0 | 0 | NA | 0.78 | 0.432 | 1.104 | ([Green et al., 2009](#_ENREF_72)) and Green et al. unpub. |
| *Alectoris graeca* | Aves | OtherBirds | Bi | 1 | 1 | 0 | 0 | NA | 0.489 | 0.690 | 1.500 | ([Fedak et al., 1974](#_ENREF_44)) |
| *Excalfactoria chinensis* | Aves | OtherBirds | Bi | 1 | 1 | 0 | 0 | NA | 0.042 | 1.200 | 2.300 | ([Fedak et al., 1974](#_ENREF_44)) |
| *Lagopus muta hyperborea* | Aves | OtherBirds | Bi | 1 | 1 | 0 | 0 | NA | 0.501 | 0.267 | 2.563 | ([Nudds et al., 2011](#_ENREF_117)) |
| *Meleagris gallopavo* | Aves | OtherBirds | Bi | 1 | 1 | 0 | 0 | NA | 4.306 | 0.410 | 0.600 | ([Fedak et al., 1974](#_ENREF_44)) |
| *Charadrius wilsonia* | Aves | OtherBirds | Bi | 1 | 1 | 0 | 0 | NA | 0.018 | 1.850 | 2.333 | ([Taylor et al., 1982b](#_ENREF_154)) |
| *Philomachus pugnax* | Aves | OtherBirds | Bi | 1 | 1 | 0 | 0 | NA | 0.11 | 0.640 | 3.244 | ([Vaillancourt et al., 2005](#_ENREF_160)) |
| *Geococcyx californianus* | Aves | OtherBirds | Bi | 1 | 1 | 0 | 0 | NA | 0.29 | 0.590 | 2.193 | ([Fedak and Seeherman, 1979](#_ENREF_45), [Taylor et al., 1982b](#_ENREF_154)) |
| *Leptoptilos crumeniferus* | Aves | OtherBirds | Bi | 1 | 1 | 0 | 0 | NA | 4.5 | 0.255 | 0.520 | ([Bamford and Maloiy, 1980](#_ENREF_5)) |
| *Morus serrator* | Aves | OtherBirds | Bi | 1 | 1 | 1 | 0 | NA | 2.31 | 0.999 | 1.457 | ([Green et al., 2013](#_ENREF_70)) and Green et al. unpub. |
| *Phalacrocorax carbo* | Aves | OtherBirds | Bi | 1 | 1 | 1 | 0 | NA | 1.98 | 1.591 | 1.341 | ([White et al., 2008a](#_ENREF_167)) |
| *Diomedea melanophrys* | Aves | OtherBirds | Bi | 1 | 1 | 1 | 0 | NA | 3.425 | 2.022 | 1.487 | ([Kabat et al., 2007](#_ENREF_93), [Bevan et al., 1994](#_ENREF_23)) |
| *Thalassarche chrysostoma* | Aves | OtherBirds | Bi | 1 | 1 | 1 | 0 | NA | 3.24 | 2.675 | 1.022 | ([Kabat et al., 2007](#_ENREF_93)) |
| *Pygoscelis adeliae* | Aves | Penguins | Bi | 1 | 1 | 1 | 0 | NA | 3.89 | 0.760 | 0.936 | ([Pinshow et al., 1977](#_ENREF_125)) |
| *Pygoscelis papua* | Aves | Penguins | Bi | 1 | 1 | 1 | 0 | NA | 6.21 | 0.605 | 1.056 | ([Bevan et al., 1995](#_ENREF_24)) |
| *Eudyptula minor* | Aves | Penguins | Bi | 1 | 1 | 1 | 0 | NA | 1.125 | 1.071 | 1.300 | ([Pinshow et al., 1977](#_ENREF_125), [Baudinette and Gill, 1985](#_ENREF_10), [Baudinette et al., 1986](#_ENREF_11)) |
| *Spheniscus magellanicus* | Aves | Penguins | Bi | 1 | 1 | 1 | 0 | NA | 3.17 | 0.699 | 0.899 | ([Halsey et al., 2009](#_ENREF_76)) and Halsey et al. unpub. |
| *Eudyptes chrysocome moselii* | Aves | Penguins | Bi | 1 | 1 | 1 | 0 | NA | 2.5 | 1.172 | 0.674 | ([Halsey et al., 2009](#_ENREF_76)) and Halsey et al. unpub. |
| *Eudyptes chrysolophus* | Aves | Penguins | Bi | 1 | 1 | 1 | 0 | NA | 3.697 | 0.595 | 0.899 | ([Green et al., 2001](#_ENREF_71)) |
| *Aptenodytes patagonicus* | Aves | Penguins | Bi | 1 | 1 | 1 | 0 | NA | 14.2 | 0.469 | 0.845 | ([Fahlman et al., 2004](#_ENREF_42)) |
| *Aptenodytes forsteri* | Aves | Penguins | Bi | 1 | 1 | 1 | 0 | NA | 23.37 | 0.406 | 0.363 | ([Pinshow et al., 1976](#_ENREF_124)) |
| *Teratoscincus przewalski* | NonAvianReptile | NonAvianReptile | Quad | 0 | 0 | 0 | 1 | 25 | 0.0112 | 1.060 | 0.165 | ([Hare et al., 2007](#_ENREF_78)) |
| *Nephrurus levis* | NonAvianReptile | NonAvianReptile | Quad | 0 | 0 | 0 | 1 | 20 | 0.0127 | 0.970 | 0.091 | ([Hare et al., 2007](#_ENREF_78)) |
| *Nephrurus asper* | NonAvianReptile | NonAvianReptile | Quad | 0 | 0 | 0 | 1 | 20 | 0.0251 | 0.762 | 0.124 | ([Hare et al., 2007](#_ENREF_78)) |
| *Diplodactylus galeatus* | NonAvianReptile | NonAvianReptile | Quad | 0 | 0 | 0 | 1 | 20 | 0.0043 | 1.689 | 0.001 | ([Hare et al., 2007](#_ENREF_78)) |
| *Diplodactylus intermedius* | NonAvianReptile | NonAvianReptile | Quad | 0 | 0 | 0 | 1 | 20 | 0.0049 | 1.434 | 0.093 | ([Hare et al., 2007](#_ENREF_78)) |
| *Naultinus manukanus* | NonAvianReptile | NonAvianReptile | Quad | 0 | 0 | 0 | 0 | 25 | 0.0066 | 0.812 | 0.013 | ([Hare et al., 2007](#_ENREF_78)) |
| *Hoplodactylus maculatus* | NonAvianReptile | NonAvianReptile | Quad | 0 | 0 | 0 | 1 | 25 | 0.006 | 0.755 | 0.149 | ([Hare et al., 2007](#_ENREF_78)) |
| *Coleonyx variegatus* | NonAvianReptile | NonAvianReptile | Quad | 0 | 0 | 0 | 1 | 25 | 0.0042 | 1.490 | 0.160 | ([Hare et al., 2007](#_ENREF_78)) |
| *Eublepharis macularius* | NonAvianReptile | NonAvianReptile | Quad | 0 | 0 | 0 | 1 | 25 | 0.0328 | 1.090 | 0.273 | ([Hare et al., 2007](#_ENREF_78)) |
| *Rhoptropus bradfieldi* | NonAvianReptile | NonAvianReptile | Quad | 0 | 0 | 0 | 0 | 25 | 0.0047 | 2.468 | 0.188 | ([Hare et al., 2007](#_ENREF_78)) |
| *Pachydactylus bibronii* | NonAvianReptile | NonAvianReptile | Quad | 0 | 0 | 0 | 1 | 25 | 0.0148 | 1.194 | 0.225 | ([Hare et al., 2007](#_ENREF_78)) |
| *Phelsuma madagascarensis* | NonAvianReptile | NonAvianReptile | Quad | 0 | 0 | 0 | 0 | 25 | 0.0239 | 1.389 | 0.151 | ([Hare et al., 2007](#_ENREF_78)) |
| *Eumeces skiltonianus* | NonAvianReptile | NonAvianReptile | Quad | 0 | 0 | 0 | 0 | 35 | 0.0048 | 2.550 | 0.350 | ([Hare et al., 2007](#_ENREF_78)) |
| *Oligosoma nigriplantare* | NonAvianReptile | NonAvianReptile | Quad | 0 | 0 | 0 | 0 | 25 | 0.0032 | 1.999 | 0.410 | ([Hare et al., 2007](#_ENREF_78)) |
| *Cyclodina macgregori* | NonAvianReptile | NonAvianReptile | Quad | 0 | 0 | 0 | 1 | 25 | 0.0202 | 0.211 | 0.072 | ([Hare et al., 2007](#_ENREF_78)) |
| *Trachydosaurus rugosus* | NonAvianReptile | NonAvianReptile | Quad | 0 | 0 | 0 | 0 | 35 | 0.474 | 0.921 | 0.107 | ([John-Alder et al., 1986](#_ENREF_91)) |
| *Dipsosaurus dorsali* | NonAvianReptile | NonAvianReptile | Quad | 0 | 0 | 0 | 0 | 25 | 0.0513 | 1.320 | 0.240 | ([John-Alder and Bennett, 1981](#_ENREF_90)) |
| *Amblyrhynchus cristatus* | NonAvianReptile | NonAvianReptile | Quad | 0 | 0 | 0 | 0 | 35 | 1.7325 | 0.561 | 0.262 | ([Gleeson, 1979](#_ENREF_68)) |
| *Conolophus subcristatus* | NonAvianReptile | NonAvianReptile | Quad | 0 | 0 | 0 | 0 | 35 | 3.885 | 0.361 | 0.309 | ([Gleeson, 1979](#_ENREF_68)) |
| *Ctenosaura similis* | NonAvianReptile | NonAvianReptile | Quad | 0 | 0 | 0 | 0 | NA | 0.126 | 1.090 | NA | ([Bakker, 1972](#_ENREF_4)) |
| *Iguana iguana* | NonAvianReptile | NonAvianReptile | Quad | 0 | 0 | 0 | 0 | NA | 1.21 | 0.950 | NA | ([Gleeson, 1979](#_ENREF_68)) |
| *Phrynosoma douglassi* | NonAvianReptile | NonAvianReptile | Quad | 0 | 0 | 0 | 0 | 35 | 0.0045 | 2.550 | 0.425 | ([Hare et al., 2007](#_ENREF_78)) |
| *Gerrhonotus spp* | NonAvianReptile | NonAvianReptile | Quad | 0 | 0 | 0 | 0 | NA | 0.014 | 1.910 | NA | ([Bakker, 1972](#_ENREF_4)) |
| *Heloderma suspectum* | NonAvianReptile | NonAvianReptile | Quad | 0 | 0 | 0 | 0 | 35 | 0.464 | 0.616 | 0.159 | ([John-Alder et al., 1983](#_ENREF_92)) |
| *Varanus species two* | NonAvianReptile | NonAvianReptile | Quad | 0 | 0 | 0 | 0 | NA | 0.3875 | 0.920 | NA | ([Bakker, 1972](#_ENREF_4)) |
| *Varanus species one* | NonAvianReptile | NonAvianReptile | Quad | 0 | 0 | 0 | 0 | NA | 0.128 | 1.550 | NA | ([Bakker, 1972](#_ENREF_4)) |
| *Varanus exanthematicus* | NonAvianReptile | NonAvianReptile | Quad | 0 | 0 | 0 | 0 | NA | 0.99 | 0.620 | NA | ([Gleeson, 1979](#_ENREF_68)) |
| *Lacerta sicula* | NonAvianReptile | NonAvianReptile | Quad | 0 | 0 | 0 | 0 | NA | 0.0082 | 2.000 | NA | ([John-Alder et al., 1986](#_ENREF_91)) |
| *Lacerta vivipara* | NonAvianReptile | NonAvianReptile | Quad | 0 | 0 | 0 | 0 | NA | 0.0034 | 3.600 | NA | ([John-Alder et al., 1986](#_ENREF_91)) |
| *Lacerta viridus* | NonAvianReptile | NonAvianReptile | Quad | 0 | 0 | 0 | 0 | NA | 0.0305 | 1.200 | NA | ([John-Alder et al., 1986](#_ENREF_91)) |
| *Cnemidophorus murinus* | NonAvianReptile | NonAvianReptile | Quad | 0 | 0 | 0 | 0 | NA | 0.0707 | 3.680 | 0.530 | ([Bennett and Gleeson, 1979](#_ENREF_19)) |
| *Tupinambis nigropunctatus* | NonAvianReptile | NonAvianReptile | Quad | 0 | 0 | 0 | 0 | 35 | 0.865 | 0.521 | 0.348 | ([Bennett and John-Alder, 1984](#_ENREF_20)) |
| *Tachyglossus aculeatus* | Mammal | Monotremata | Quad | 0 | 1 | 0 | 0 | NA | 5.04 | 0.300 | 0.350 | ([Crompton et al., 1978](#_ENREF_32)) |
| *Ornithorhynchus anatinus* | Mammal | Monotremata | Quad | 0 | 1 | 0 | 0 | NA | 1.4 | 3.672 | 0.450 | ([Fish et al., 2001](#_ENREF_49)) |
| *Pedetes capensis* | Mammal | Rodentia | Quad | 0 | 1 | 0 | 0 | NA | 3 | 0.340 | 2.052 | ([Seeherman et al., 1981](#_ENREF_146)) |
| *Notomys alexis* | Mammal | Rodentia | Quad | 0 | 1 | 0 | 0 | NA | 0.0274 | 1.960 | 3.210 | ([Baudinette et al., 1976a](#_ENREF_14)) |
| *Notomys cervinus* | Mammal | Rodentia | Quad | 0 | 1 | 0 | 0 | NA | 0.036 | 0.770 | 3.250 | ([Dawson, 1976](#_ENREF_33)) |
| *Mus musculus* | Mammal | Rodentia | Quad | 0 | 1 | 0 | 0 | NA | 0.0295 | 2.004 | 3.584 | ([Taylor et al., 1970b](#_ENREF_157), [Taylor et al., 1972](#_ENREF_152), [Oron et al., 1981](#_ENREF_119), [Rezende et al., 2006](#_ENREF_134)) |
| *Rattus rattus* | Mammal | Rodentia | Quad | 0 | 1 | 0 | 0 | NA | 0.256 | 0.770 | 1.940 | ([Lawler et al., 1993](#_ENREF_99)) |
| *Rattus norvegicus* | Mammal | Rodentia | Quad | 0 | 1 | 0 | 0 | NA | 0.205 | 0.970 | 1.980 | ([Seeherman et al., 1981](#_ENREF_146)) |
| *Baiomys taylori* | Mammal | Rodentia | Quad | 0 | 1 | 0 | 0 | NA | 0.0072 | 2.250 | 3.348 | ([Seeherman et al., 1981](#_ENREF_146)) |
| *Meriones unguiculatus* | Mammal | Rodentia | Quad | 0 | 1 | 0 | 0 | NA | 0.0677 | 0.971 | 1.937 | ([Chappell et al., 2007](#_ENREF_29)) |
| *Dipodomys merriami* | Mammal | Rodentia | Quad | 0 | 1 | 0 | 0 | NA | 0.0347 | 2.803 | 2.930 | ([Taylor et al., 1970b](#_ENREF_157), [Thompson et al., 1980](#_ENREF_159), [Yousef et al., 1970](#_ENREF_175)) |
| *Dipodomys deserti* | Mammal | Rodentia | Quad | 0 | 1 | 0 | 0 | NA | 0.104 | 0.660 | 3.250 | ([Thompson et al., 1980](#_ENREF_159)) |
| *Dipodomys spectabilis* | Mammal | Rodentia | Quad | 0 | 1 | 0 | 0 | NA | 0.1 | 1.130 | 1.810 | ([Taylor et al., 1970b](#_ENREF_157)) |
| *Myocastor coypus* | Mammal | Rodentia | Quad | 0 | 1 | 0 | 0 | NA | 2.945 | 0.532 | 0.767 | ([Halsey et al., 2009](#_ENREF_76)) and Halsey et al. unpub. |
| *Spermophilus tereticaudus* | Mammal | Rodentia | Quad | 0 | 1 | 0 | 0 | NA | 0.236 | 0.660 | 1.270 | ([Taylor et al., 1970b](#_ENREF_157)) |
| *Spermophilus saturatus* | Mammal | Rodentia | Quad | 0 | 1 | 0 | 0 | NA | 0.23 | 0.697 | 3.860 | ([Hoyt and Kenagy, 1988](#_ENREF_85)) |
| *Ammospermophilus leucurus* | Mammal | Rodentia | Quad | 0 | 1 | 0 | 0 | NA | 0.0186 | 1.417 | 3.300 | ([Yousef et al., 1973](#_ENREF_176)) |
| *Tamias striatus* | Mammal | Rodentia | Quad | 0 | 1 | 0 | 0 | NA | 0.0902 | 0.780 | 2.304 | ([Seeherman et al., 1981](#_ENREF_146)) |
| *Tamias merriami* | Mammal | Rodentia | Quad | 0 | 1 | 0 | 0 | NA | 0.075 | 1.800 | 1.690 | ([Wunder, 1970](#_ENREF_173)) |
| *Tamiasciurus hudsonicus* | Mammal | Rodentia | Quad | 0 | 1 | 0 | 0 | NA | 0.252 | 0.780 | 2.320 | ([Wunder and Morrison, 1974](#_ENREF_174)) |
| *Glaucomys volans* | Mammal | Rodentia | Quad | 0 | 1 | 0 | 0 | NA | 0.063 | 1.200 | 2.340 | ([Taylor et al., 1982b](#_ENREF_154)) |
| *Glaucomys sabrinus* | Mammal | Rodentia | Quad | 0 | 1 | 0 | 0 | NA | 0.137 | 2.320 | NA | ([Flaherty et al., 2010](#_ENREF_50)) |
| *Erythrocebus patas* | Mammal | Euarchonta | Quad | 0 | 1 | 0 | 0 | NA | 3.8 | 0.255 | 0.352 | ([Mahoney, 1980](#_ENREF_110)) |
| *Papio hamadryas* | Mammal | Euarchonta | Quad | 0 | 1 | 0 | 0 | NA | 8.5 | 0.240 | 1.296 | ([Taylor et al., 1982b](#_ENREF_154)) |
| *Macaca arctoides* | Mammal | Euarchonta | Quad | 0 | 1 | 0 | 0 | NA | 5.1 | 0.250 | 1.548 | ([Taylor et al., 1982b](#_ENREF_154)) |
| *Macaca fuscata* | Mammal | Euarchonta | Quad | 0 | 1 | 0 | 0 | NA | 9 | 0.404 | 0.749 | ([Nakatsukasa et al., 2004](#_ENREF_115)) |
| *Homo sapiens* | Mammal | Euarchonta | Bi | 1 | 1 | 0 | 0 | NA | 65 | 0.150 | 0.700 | ([Halsey and White, 2012](#_ENREF_77)) |
| *Pan troglodytes* | Mammal | Euarchonta | Quad | 0 | 1 | 0 | 0 | NA | 17.5 | 0.250 | 0.790 | ([Taylor et al., 1972](#_ENREF_152)) |
| *Ateles geoffroyi* | Mammal | Euarchonta | Quad | 0 | 1 | 0 | 0 | NA | 4.1 | 0.370 | 0.840 | ([Parsons and Taylor, 1977](#_ENREF_123)) |
| *Cebus capucinus* | Mammal | Euarchonta | Quad | 0 | 1 | 0 | 0 | NA | 3.34 | 0.280 | 1.500 | ([Taylor and Rowntree, 1973](#_ENREF_155)) |
| *Nycticebus coucang* | Mammal | Euarchonta | Quad | 0 | 1 | 0 | 0 | NA | 1.215 | 0.580 | 0.800 | ([Parsons and Taylor, 1977](#_ENREF_123)) |
| *Galago senegalensis* | Mammal | Euarchonta | Quad | 0 | 1 | 0 | 0 | NA | 0.24 | 1.470 | 1.728 | ([Taylor et al., 1982b](#_ENREF_154)) |
| *Tupaia glis* | Mammal | Euarchonta | Quad | 0 | 1 | 0 | 0 | NA | 0.124 | 0.690 | 1.620 | ([Taylor et al., 1982b](#_ENREF_154)) |
| *Connochaetes taurinus* | Mammal | Artiodactyla | Quad | 0 | 1 | 0 | 0 | NA | 92 | 0.092 | 0.248 | ([Taylor et al., 1982b](#_ENREF_154)) |
| *Ovis aries* | Mammal | Artiodactyla | Quad | 0 | 1 | 0 | 0 | NA | 23 | 0.230 | 0.360 | ([Taylor et al., 1982b](#_ENREF_154)) |
| *Capra hircus* | Mammal | Artiodactyla | Quad | 0 | 1 | 0 | 0 | NA | 24 | 0.220 | 0.391 | ([Taylor et al., 1974](#_ENREF_158), [Taylor et al., 1982b](#_ENREF_154)) |
| *Gazella gazella* | Mammal | Artiodactyla | Quad | 0 | 1 | 0 | 0 | NA | 23.15 | 0.160 | 0.550 | ([Taylor et al., 1974](#_ENREF_158)) |
| *Kobus ellipsiprymnus* | Mammal | Artiodactyla | Quad | 0 | 1 | 0 | 0 | NA | 114 | 0.130 | 0.166 | ([Taylor et al., 1982b](#_ENREF_154)) |
| *Madoqua kirkii* | Mammal | Artiodactyla | Quad | 0 | 1 | 0 | 0 | NA | 4.35 | 0.400 | 0.230 | ([Taylor et al., 1982b](#_ENREF_154)) |
| *Neotragus moschatus* | Mammal | Artiodactyla | Quad | 0 | 1 | 0 | 0 | NA | 3.5 | 0.520 | 0.612 | ([Taylor et al., 1982b](#_ENREF_154)) |
| *Bos taurus* | Mammal | Artiodactyla | Quad | 0 | 1 | 0 | 0 | NA | 254 | 0.096 | 0.094 | ([Taylor et al., 1982b](#_ENREF_154)) |
| *Taurotragus oryx* | Mammal | Artiodactyla | Quad | 0 | 1 | 0 | 0 | NA | 213 | 0.083 | 0.259 | ([Taylor et al., 1982b](#_ENREF_154)) |
| *Rangifer tarandus* | Mammal | Artiodactyla | Quad | 0 | 1 | 0 | 0 | NA | 54 | 0.090 | 0.041 | ([Luick and White, 1986](#_ENREF_109), [Fancy and White, 1985](#_ENREF_43)) |
| *Cervus elaphus* | Mammal | Artiodactyla | Quad | 0 | 1 | 0 | 0 | NA | 42 | 0.229 | 0.496 | ([Parker et al., 1984](#_ENREF_122)) |
| *Sus scrofa* | Mammal | Artiodactyla | Quad | 0 | 1 | 0 | 0 | NA | 18.5 | 0.290 | 0.468 | ([Seeherman et al., 1981](#_ENREF_146)) |
| *Camelus dromedarius* | Mammal | Artiodactyla | Quad | 0 | 1 | 0 | 0 | NA | 240 | 0.056 | 0.108 | ([Maloiy et al., 2009](#_ENREF_111)) |
| *Equus asinus* | Mammal | Perissodactyla | Quad | 0 | 1 | 0 | 0 | NA | 170 | 0.074 | 0.176 | ([Maloiy et al., 2009](#_ENREF_111)) |
| *Equus caballus* | Mammal | Perissodactyla | Quad | 0 | 1 | 0 | 0 | NA | 467 | 0.128 | 1.040 | ([Wickler et al., 2003](#_ENREF_169)) |
| *Mustela nigripes* | Mammal | Carnivora | Quad | 0 | 1 | 0 | 0 | NA | 0.542 | 0.520 | 2.016 | ([Taylor et al., 1982b](#_ENREF_154)) |
| *Mustela vison* | Mammal | Carnivora | Quad | 0 | 1 | 0 | 0 | NA | 0.868 | 0.510 | 2.700 | ([Williams, 1983](#_ENREF_170)) |
| *Lontra canadensis* | Mammal | Carnivora | Quad | 0 | 1 | 0 | 0 | NA | 11.1 | 0.390 | 0.846 | ([Williams et al., 2002](#_ENREF_171)) |
| *Conepatus chinga* | Mammal | Carnivora | Quad | 0 | 1 | 0 | 0 | NA | 3.1 | 0.878 | 0.442 | ([Halsey et al., 2009](#_ENREF_76)) and Halsey et al. unpub. |
| *Ursus maritimus* | Mammal | Carnivora | Quad | 0 | 1 | 0 | 0 | NA | 190 | 0.220 | 0.205 | ([Hurst et al., 1982](#_ENREF_86)) |
| *Lycaon pictus* | Mammal | Carnivora | Quad | 0 | 1 | 0 | 0 | NA | 8.75 | 0.294 | 1.046 | ([Taylor et al., 1971b](#_ENREF_156)) |
| *Alopex lagopus* | Mammal | Carnivora | Quad | 0 | 1 | 0 | 0 | NA | 3.855 | 0.146 | 1.466 | ([Fuglei and Øritsland, 2003](#_ENREF_53)) |
| *Panthera leo* | Mammal | Carnivora | Quad | 0 | 1 | 0 | 0 | NA | 53.5 | 0.360 | NA | ([Chassin et al., 1976](#_ENREF_30)) |
| *Felis silvestris* | Mammal | Carnivora | Quad | 0 | 1 | 0 | 0 | NA | 3.9 | 0.400 | 0.155 | ([Taylor et al., 1982b](#_ENREF_154)) |
| *Acinonyx jubatus* | Mammal | Carnivora | Quad | 0 | 1 | 0 | 0 | NA | 39 | 0.140 | 0.700 | ([Taylor et al., 1974](#_ENREF_158)) |
| *Helogale parvula* | Mammal | Carnivora | Quad | 0 | 1 | 0 | 0 | NA | 0.583 | 0.670 | 1.296 | ([Taylor et al., 1982b](#_ENREF_154)) |
| *Mungos mungo* | Mammal | Carnivora | Quad | 0 | 1 | 0 | 0 | NA | 1.15 | 0.420 | 1.476 | ([Taylor et al., 1982b](#_ENREF_154)) |
| *Genetta tigrina* | Mammal | Carnivora | Quad | 0 | 1 | 0 | 0 | NA | 1.46 | 0.640 | 1.368 | ([Taylor et al., 1982b](#_ENREF_154)) |
| *Erinaceus europaeus* | Mammal | Eulipotyphla | Quad | 0 | 1 | 0 | 0 | NA | 1.05 | 0.460 | 0.480 | ([Crompton et al., 1978](#_ENREF_32)) |
| *Suncus murinus* | Mammal | Eulipotyphla | Quad | 0 | 1 | 0 | 0 | NA | 0.036 | 1.620 | 3.430 | ([Oron et al., 1981](#_ENREF_119)) |
| *Chaetophractus villosus* | Mammal | Xenarthra | Quad | 0 | 1 | 0 | 0 | NA | 2.98 | 0.451 | 0.776 | ([Halsey et al., 2009](#_ENREF_76)) and Halsey et al. unpub. |
| *Dasypus novemcinctus* | Mammal | Xenarthra | Quad | 0 | 1 | 0 | 0 | NA | 4.072 | 0.250 | 0.660 | ([Oron et al., 1981](#_ENREF_119)) |
| *Tenrec ecaudatus* | Mammal | Afrotheria | Quad | 0 | 1 | 0 | 0 | NA | 0.695 | 0.580 | 0.390 | ([Crompton et al., 1978](#_ENREF_32)) |
| *Setifer setosus* | Mammal | Afrotheria | Quad | 0 | 1 | 0 | 0 | NA | 0.12 | 0.680 | 0.370 | ([Crompton et al., 1978](#_ENREF_32)) |
| *Elephantulus rufescens* | Mammal | Afrotheria | Quad | 0 | 1 | 0 | 0 | NA | 0.06 | 1.650 | 1.930 | ([Oron et al., 1981](#_ENREF_119)) |
| *Elephas maximus* | Mammal | Afrotheria | Quad | 0 | 1 | 0 | 0 | NA | 3113.5 | 0.068 | NA | ([Langman et al., 2012](#_ENREF_98)) |
| *Loxodonta africana* | Mammal | Afrotheria | Quad | 0 | 1 | 0 | 0 | NA | 1542 | 0.036 | 0.054 | ([Langman et al., 1995](#_ENREF_97)) |
| *Trichosurus vulpecula* | Mammal | Metatheria | Quad | 0 | 1 | 0 | 0 | NA | 3.9 | 0.440 | 1.310 | ([Baudinette et al., 1978](#_ENREF_16)) |
| *Bettongia penicillata* | Mammal | Metatheria | Quad | 0 | 1 | 0 | 0 | NA | 1.1 | 0.390 | 1.728 | ([Seeherman et al., 1981](#_ENREF_146)) |
| *Macropus eugenii* | Mammal | Metatheria | Quad | 0 | 1 | 0 | 0 | NA | 4.9 | 0.340 | 0.540 | ([Baudinette et al., 1992](#_ENREF_17)) |
| *Macropus rufus* | Mammal | Metatheria | Quad | 0 | 1 | 0 | 0 | NA | 18 | 0.660 | 0.350 | ([Dawson and Taylor, 1973](#_ENREF_34)) |
| *Setonix brachyurus* | Mammal | Metatheria | Quad | 0 | 1 | 0 | 0 | NA | 3 | 0.380 | 2.180 | ([Baudinette, 1977](#_ENREF_9)) |
| *Potorous tridactylus* | Mammal | Metatheria | Quad | 0 | 1 | 0 | 0 | NA | 1.084 | 0.364 | 1.278 | ([Baudinette et al., 1993](#_ENREF_12)) |
| *Antechinus flavipes* | Mammal | Metatheria | Quad | 0 | 1 | 0 | 0 | NA | 0.041 | 1.555 | 5.835 | ([Baudinette et al., 1976b](#_ENREF_15)) |
| *Dasycercus byrnei* | Mammal | Metatheria | Quad | 0 | 1 | 0 | 0 | NA | 0.115 | 0.840 | 2.860 | ([Baudinette et al., 1976b](#_ENREF_15)) |
| *Dasyurus viverrinus* | Mammal | Metatheria | Quad | 0 | 1 | 0 | 0 | NA | 1.12 | 0.430 | 2.430 | ([Baudinette et al., 1976b](#_ENREF_15)) |
| *Sminthopsis crassicaudata* | Mammal | Metatheria | Quad | 0 | 1 | 0 | 0 | NA | 0.015 | 1.620 | 6.830 | ([Baudinette et al., 1976b](#_ENREF_15)) |
| *Antechinomys laniger* | Mammal | Metatheria | Quad | 0 | 1 | 0 | 0 | NA | 0.02995 | 1.470 | 4.310 | ([Baudinette et al., 1976a](#_ENREF_14)) |
| *Didelphis virginiana* | Mammal | Metatheria | Quad | 0 | 1 | 0 | 0 | NA | 2.14 | 0.350 | 1.016 | ([Crompton et al., 1978](#_ENREF_32), [Fournier and Weber, 1994](#_ENREF_52)) |
| *Monodelphis domestica* | Mammal | Metatheria | Quad | 0 | 1 | 0 | 0 | NA | 0.068 | 1.690 | 1.580 | ([Oron et al., 1981](#_ENREF_119)) |

**
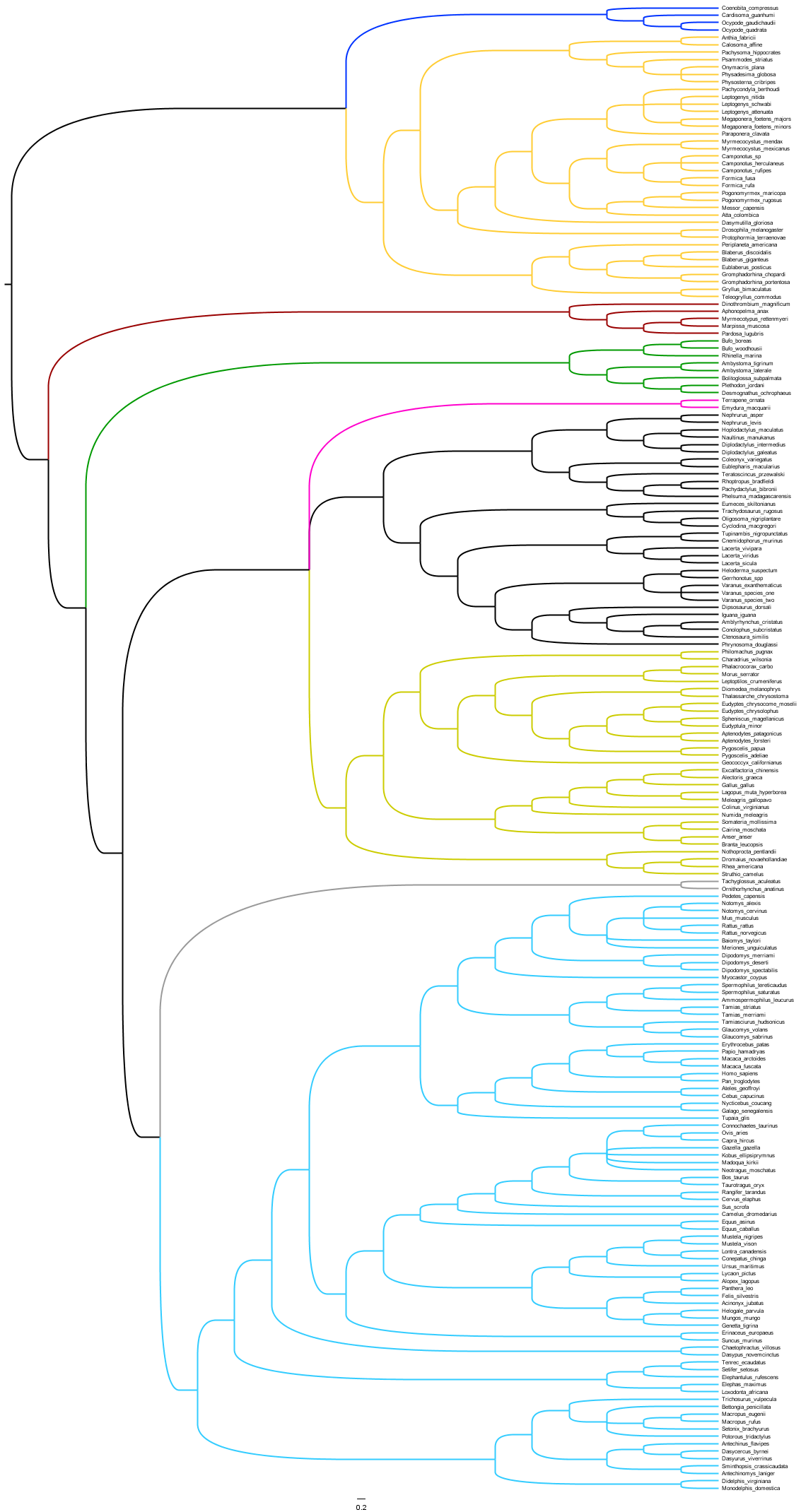
**

**Appendix – References**

AUTUMN, K., FARLEY, C. T., EMSHWILLER, M. & FULL, R. J. (1997) Low cost of locomotion in the banded gecko: A test of the nocturnality hypothesis. *Physiological Zoology,* 70**,** 660-669.

AUTUMN, K., JINDRICH, D., DENARDO, D. & MUELLER, R. (1999) Locomotor performance at low temperature and the evolution of nocturnality in geckos. *Evolution,* 53**,** 580-599.

AUTUMN, K., WEINSTEIN, R. B. & FULL, R. J. (1994) Low cost of locomotion increases performance at low temperature in a nocturnal lizard. *Physiological Zoology,* 67**,** 238-262.

BAKKER, R. T. (1972) Locomotor energetics of lizards and mammals compared. *Physiologist,* 15**,** 76.

BAMFORD, O. S. & MALOIY, G. M. O. (1980) Energy metabolism and heart rate during treadmill exercise in the Marabou stork. *Journal of Applied Physiology,* 49**,** 491-496.

BÁRÁNY, M. (1967) ATPase activity of myosin correlated with speed of muscle shortening. *The Journal of General Physiology,* 50**,** 197-218.

BARTHOLOMEW, G. A. & LIGHTON, J. R. B. (1985) Ventilation and oxygen consumption during rest and locomotion in a tropical cockroach, *Blaberus giganteus*. *Journal of Experimental Biology,* 118**,** 449-454.

BARTHOLOMEW, G. A., LIGHTON, J. R. B. & LOUW, G. N. (1985) Energetics of locomotion and patterns of respiration in tenebrionid beetles from the Namib Desert (South Africa). *Journal of Comparative Physiology B,* 155**,** 155-162.

BAUDINETTE, R. V. (1977) Locomotory energetics in a marsupial, *Setonix Brachyurus*. *Australian Journal of Zoology,* 25**,** 423-428.

BAUDINETTE, R. V. & GILL, P. (1985) The energetics of ‘flying’ and ‘paddling’ in water: locomotion in penguins and ducks. *Journal of Comparative Physiology B,* 155**,** 373-380.

BAUDINETTE, R. V., GILL, P. & O'DRISCOLL, M. (1986) Energetics of the little penguin, *Eudyptula minor*: temperature regulation, the calorigenic effect of food, and molting. *Australian Journal of Zoology,* 34**,** 35-46.

BAUDINETTE, R. V., HALPERN, E. A. & HINDS, D. S. (1993) Energetic cost of locomotion as a function of ambient temperature and during growth in the marsupial *Potorous tridactylus*. *Journal of Experimental Biology,* 174**,** 81-95.

BAUDINETTE, R. V., MILLER, A. M. & SARRE, M. P. (2000) Aquatic and terrestrial locomotory energetics in a toad and a turtle: A search for generalisations among ectotherms. *Physiological and Biochemical Zoology,* 73**,** 672-682.

BAUDINETTE, R. V., NAGLE, K. A. & SCOTT, R. A. D. (1976a) Locomotory energetics in a marsupial (*Antechinomys spenceri*) and a rodent (*Notomys alexis*). *Experentia,* 32**,** 583-585.

BAUDINETTE, R. V., NAGLE, K. A. & SCOTT, R. A. D. (1976b) Locomotory energetics in dasyurid marsupials. *Journal of Comparative Physiology B: Biochemical, Systemic, and Environmental Physiology,* 109**,** 159-168.

BAUDINETTE, R. V., SEYMOUR, R. S. & ORBACH, J. (1978) Cardiovascular responses to exercise in the brush-tailed possum. *Journal of Comparative Physiology B: Biochemical, Systemic, and Environmental Physiology,* 124**,** 143-147.

BAUDINETTE, R. V., SNYDER, G. K. & FRAPPELL, P. B. (1992) Energetic cost of locomotion in the tammar wallaby. *American Journal of Physiology,* 262**,** R771-R778.

BENNETT, A. F. (1982) Energetics of activity in reptiles. IN GANS, C. & POUGH, F. H. (Eds.) *Biology of the Reptilia.* New York, Academic Press.

BENNETT, A. F. & GLEESON, T. T. (1979) Metabolic expenditure and the cost of foraging in the lizard *Cnemidophorus murinus*. *Copeia,* 1979**,** 573-577.

BENNETT, A. F. & JOHN-ALDER, H. B. (1984) The effect of body temperature on the locomotory energetics of lizards. *Journal of Comparative Physiology B,* 155**,** 21-27.

BERRIGAN, D. & LIGHTON, J. R. B. (1994) Energetics of pedestrian locomotion in adult male blowflies, *Protophormia terraenovae* (Diptera: Calliphoridae). *Physiological Zoology,* 67**,** 1140-1153.

BERRIGAN, D. & PATRIDGE, L. (1997) Influence of temperature and activity on the metabolic rate of adult *Drosophila melanogaster*. *Comparative Biochemistry and Physiology A,* 118**,** 1301-1307.

BEVAN, R. M., WOAKES, A. J., BUTLER, P. J. & BOYD, I. L. (1994) The use of heart rate to estimate oxygen consumption of free-ranging black-browed albatrosses *Diomedea melanophrys*. *Journal of Experimental Biology,* 193**,** 119-137.

BEVAN, R. M., WOAKES, A. J., BUTLER, P. J. & CROXALL, J. P. (1995) Heart rate and oxygen consumption of exercising Gentoo penguins. *Physiological Zoology,* 68**,** 855-877.

BININDA-EMONDS, O. R. P., CARDILLO, M., JONES, K. E., MACPHEE, R. D. E., BECK, R. M. D., GRENYER, R., PRICE, S. A., VOS, R. A., GITTLEMAN, J. L. & PURVIS, A. (2007) The delayed rise of present-day mammals. *Nature,* 446**,** 507-512.

BROWNING, R. C., BAKER, E. A., HERRON, J. A. & KRAM, R. (2006) Effects of obesity and sex on the energetic cost and preferred speed of walking. *Journal of Applied Physiology,* 100**,** 390-398.

BURNHAM, K. P. & ANDERSON, D. R. (2010) *Model selection and multi-model inference: a practical information-theoretic approach,* New York, Springer.

CALDER, W. A., III (1984) *Size, function, and life history,* Cambridge, Harvard University Press.

CHAPPELL, M. A., GARLAND, T., JR, ROBERTSON, G. & SALTZMAN, W. (2007) Relationships among running performance, aerobic physiology and organ mass in male Mongolian gerbils. *Journal of Experimental Biology,* 210**,** 4179-4197.

CHASSIN, P. S., TAYLOR, C. R., HEGLUND, N. C. & SEEHERMAN, H. J. (1976) Locomotion in lions: energetic cost and maximum aerobic capacity. *Physiological Zoology,* 49**,** 1-10.

CLUSELLA-TRULLAS, S., TERBLANCHE, J. S. & CHOWN, S. L. (2010) Phenotypic plasticity of locomotion performance in the seed harvester *Messor capensis* (Formicidae). *Physiological and Biochemical Zoology,* 83**,** 519-530.

CROMPTON, A. W., TAYLOR, C. R. & JAGGER, J. A. (1978) Evolution of homeothermy in mammals. *Nature,* 272**,** 333-336.

DAWSON, T. J. (1976) Energetic cost of locomotion in Australian hopping mice. *Nature,* 259**,** 305-307.

DAWSON, T. J. & TAYLOR, C. R. (1973) Energetic cost of locomotion in kangaroos. *Nature,* 246**,** 313-314.

DELL, A. I., PAWAR, S. & SAVAGE, V. M. (2011) Systematic variation in the temperature dependence of physiological and ecological traits. *Proceedings of The National Academy of Sciences of The United States of America,* 108**,** 10591-10596.

DUNCAN, F. D. (1995) A reason for division of labor in ant foraging. *Naturwissenschaften,* 82**,** 293-296.

DUNCAN, F. D. (1999) The ponerine ant *Pachycondyla* (=*Ophthalmopone*) *berthoudi* Forel carries loads economically. *Physiological and Biochemical Zoology,* 72**,** 71-77.

DUNCAN, F. D. & CREWE, R. M. (1993) A comparison of the energetics of foraging of three species of *Leptogenys* (Hymenoptera, Formicidae). *Physiological Entomology,* 18**,** 372-378.

DUNCAN, F. D. & LIGHTON, J. R. B. (1994) The burden within: The energy cost of load carriage in the honeypot ant, *Myrmecocystus*. *Physiological Zoology,* 67**,** 190-203.

DUNCAN, F. D. & LIGHTON, J. R. B. (1997) Discontinuous ventilation and energetics of locomotion in the desert-dwelling female mutillid wasp, *Dasymutilla gloriosa*. *Physiological Entomology,* 22**,** 310-315.

ELLERBY, D. J., CLEARY, M., MARSH, R. L. & BUCHANAN, C. I. (2003) Measurement of maximum oxygen consumption in Guinea fowl *Numida meleagris* indicates that birds and mammals display a similar diversity of aerobic scopes during running. *Physiological and Biochemical Zoology,* 76**,** 695-703.

FAHLMAN, A., HANDRICH, Y., WOAKES, A. J., BOST, C. A., HOLDER, R., DUCHAMP, C. & BUTLER, P. J. (2004) Effect of fasting on the VO2-f(h) relationship in king penguins, *Aptenodytes patagonicus*. *American Journal of Physiology-Regulatory Integrative and Comparative Physiology,* 287**,** R870-R877.

FANCY, S. G. & WHITE, R. G. (1985) Incremental cost of activity. IN HUDSON, R. J. & WHITE, R. G. (Eds.) *Bioenergetics of wild herbivores.* Boca Raton, CRC Pess.

FEDAK, M. A., PINSHOW, B. & SCHMIDT-NIELSEN, K. (1974) Energy cost of bipedal running. *American Journal of Physiology,* 227**,** 1038-1044.

FEDAK, M. A. & SEEHERMAN, H. J. (1979) Reappraisal of energetics of locomotion shows identical costs in bipeds and quadrupeds including ostrich and horse. *Nature,* 282**,** 713-716.

FEDER, M. E. (1986) Effect of thermal acclimation on locomotor energetics and locomotor performance in a lungless salamander, *Desmognathus ochrophaeus*. *Journal of Experimental Biology,* 121**,** 271-283.

FEDER, M. E. (1987) Effect of thermal acclimation on locomotor energetics and locomotor performance in a tropical salamander, *Bolitoglossa subpalmata*. *Physiological Zoology,* 60**,** 18-26.

FEWELL, J. H., HARRISON, J. F., LIGHTON, J. R. B. & BREED, M. D. (1996) Foraging energetics of the ant, *Paraponera clavata*. *Oecologia,* 105**,** 419-427.

FISH, F. E., FRAPPELL, P. B., BAUDINETTE, R. V. & MACFARLANE, P. M. (2001) Energetics of terrestrial locomotion of the platypus *Ornithorhynchus anatinus*. *Journal of Experimental Biology,* 204**,** 797-803.

FLAHERTY, E., BEN-DAVID, M. & SMITH, W. P. (2010) Quadrupedal locomotor performance in two species of arboreal squirrels: predicting energy savings of gliding. *Journal of Comparative Physiology B: Biochemical, Systemic, and Environmental Physiology,* 180**,** 1067-1078.

FLEMING, P. A. & BATEMAN, P. W. (2007) Just drop it and run: the effect of limb autotomy on running distance and locomotion energetics of field crickets (*Gryllus bimaculatus*). *Journal of Experimental Biology,* 210**,** 1446-1454.

FOURNIER, R. A. & WEBER, J.-M. (1994) Locomotory energetics and metabolic fuel reserves of the Virginia opossum. *Journal of Experimental Biology,* 197**,** 1-16.

FUGLEI, E. & ØRITSLAND, N. A. (2003) Energy cost of running in an Arctic fox, *Alopex lagopus*. *Canadian Field-Naturalist,* 117**,** 430-435.

FULL, R., ZUCCARELLO, D. & TULLIS, A. (1990a) Effect of variation in form on the cost of terrestrial locomotion. *J Exp Biol,* 150**,** 233-246.

FULL, R. J. (1986) Locomotion without lungs: energetics and performance of a lungless salamander. *American Journal of Physiology - Regulatory, Integrative and Comparative Physiology,* 251**,** R775-R780.

FULL, R. J. (1987) Locomotion Energetics of the Ghost Crab I. Metabolic Cost and Endurance. *Journal of Experimental Biology,* 130**,** 137-154.

FULL, R. J. (1989) Mechanics and energetics of terrestrial locomotion: From bipeds to polypeds. IN WIESER, W. & GNAIGER, E. (Eds.) *Energy transformations in cells and animals.* Stuttgart, Georg Thieme Verlag.

FULL, R. J., ANDERSON, B. D., FINNERTY, C. M. & FEDER, M. E. (1988) Exercising with and without lungs: I. The effects of metabolic cost, maximal oxygen transport and body size on terrestrial locomotion in salamander species. *Journal of Experimental Biology,* 138**,** 471-485.

FULL, R. J. & HERREID, C. F. (1983) Aerobic response to exercise of the fastest land crab. *American Journal of Physiology - Regulatory, Integrative and Comparative Physiology,* 244**,** R530-R536.

FULL, R. J. & TULLIS, A. (1990) Energetics of ascent: Insects on inclines. *Journal of Experimental Biology,* 149**,** 307-318.

FULL, R. J., ZUCCARELLO, D. A. & TULLIS, A. (1990b) Effect of variation in form on the cost of terrestrial locomotion. *Journal of Experimental Biology,* 150**,** 233-246.

GALIPAUD, M., GILLINGHAM, M. A. F., DAVID, M. & DECHAUME-MONCHARMONT, F.-X. (2014) Ecologists overestimate the importance of predictor variables in model averaging: a plea for cautious interpretations. *Methods in Ecology and Evolution,* 5**,** 983-991.

GARLAND, T., JR & ADOLPH, S. C. (1994) Why not to do two species comparative studies: limitations on inferring adaptation. *Physiological and Biochemical Zoology,* 67**,** 797-828.

GARLAND, T., JR. (1983) Scaling the ecological cost of transport to body mass in terrestrial mammals. *American Naturalist,* 121**,** 571-587.

GARLAND, T., JR. & IVES, A. R. (2000) Using the past to predict the present: Confidence intervals for regression equations in phylogenetic comparative methods. *American Naturalist,* 155**,** 346-364.

GATESY, S. & BIEWENER, A. (1991) Bipedal locomotion: effects of speed, size and limb posture in birds and humans. *Journal of Zoology (London),* 224**,** 127-147.

GEFEN, E. (2011) The relative importance of respiratory water loss in scorpions Is correlated with species habitat type and activity pattern. *Physiological and Biochemical Zoology,* 84**,** 68-76.

GLEESON, T. T. (1979) The effects of training and capacity on the metabolic capacity of the lizard *Sceloporus occidentalis*. *Journal of Comparative Physiology,* 129**,** 123-128.

GRAFEN, A. (1989) The phylogenetic regression. *Philosophical Transactions of the Royal Society of London B,* 326**,** 119-157.

GREEN, J. A., AITKEN-SIMPSON, E. J., WHITE, C. R., BUNCE, A., BUTLER, P. J. & FRAPPELL, P. B. (2013) An increase in minimum metabolic rate and not activity explains field metabolic rate changes in a breeding seabird. *Journal of Experimental Biology,* 216**,** 1726-1735.

GREEN, J. A., BUTLER, P. J., WOAKES, A. J., BOYD, I. L. & HOLDER, R. L. (2001) Heart rate and rate of oxygen consumption of expercising macaroni penguins. *Journal of Experimental Biology,* 204**,** 673-684.

GREEN, J. A., HALSEY, L. G., WILSON, R. P. & FRAPPELL, P. B. (2009) Estimating energy expenditure of animals using the accelerometry technique: activity, inactivity and comparison with the heart-rate technique. *Journal of Experimental Biology,* 212**,** 471-482.

GRIFFIN, T. M. & KRAM, R. (2000) Penguin waddling is not wasteful. *Nature,* 408**,** 929.

HALSEY, L. G. (2013) The relationship between energy expenditure and speed during pedestrian locomotion in birds: A morphological basis for the elevated y-intercept? *Comparative Biochemistry and Physiology A,* 165**,** 295-298.

HALSEY, L. G., MATTHEWS, P. G. D., REZENDE, E. L., CHAUVAUD, L. & ROBSON, A. A. (2015) The interactions between temperature and activity levels in driving metabolic rate: theory, with empirical validation from contrasting ectotherms. *Oecologia,* 177**,** 1117-1129.

HALSEY, L. G., SHEPARD, E. L. C., QUINTANA, F., LAICH, A. G., GREEN, J. A. & WILSON, R. P. (2009) The relationship between oxygen consumption and body acceleration in a range of species. *Comparative Biochemistry and Physiology A,* 152**,** 197-202.

HALSEY, L. G. & WHITE, C. R. (2012) Comparative energetics of mammalian locomotion: Humans are not different. *Journal of Human Evolution,* 63**,** 718-722.

HARE, K. M., PLEDGER, S., THOMPSON, M. B., MILLER, J. H. & DAUGHERTY, C. H. (2007) Low cost of locomotion in lizards that are active at low temperatures. *Physiological and Biochemical Zoology,* 80**,** 46-58.

HAWKINS, P. A. J., BUTLER, P. J., WOAKES, A. J. & SPEAKMAN, J. R. (2000) Estimation of the rate of oxygen consumption of the common eider duck (*Somateria mollissima*), with some measurements of heart rate during voluntary dives. *Journal of Experimental Biology,* 203**,** 2819-2832.

HEGLUND, N. C. & TAYLOR, C. R. (1988) Speed, stride, frequency and energy cost per stride: how do they change with body size and gait? *Journal of Experimental Biology,* 138**,** 301-318.

HERREID, C. F. & FULL, R. J. (1984) Cockroaches on a treadmill: aerobic running. *Journal of Insect Physiology,* 30**,** 395-403.

HERREID, C. F. & FULL, R. J. (1986) Energetics of hermit crabs (*Coenobita compressus*) during locomotion: The cost of carrying a shell. *Journal of Experimental Biology,* 120**,** 297-308.

HERREID, C. F., FULL, R. J. & PRAWEL, D. A. (1981) Energetics of cockroach locomotion. *Journal of Experimental Biology,* 94**,** 189-202.

HERREID, C. F., LEE, L. W. & SHAH, G. M. (1979) Respiration and heart rate in exercising land crabs. *Respiration Physiology,* 37**,** 109-120.

HOYT, D. F. & KENAGY, G. J. (1988) Energy costs of walking and running gaits and their aerobic limits in golden-mantled ground squirrels. *Physiological Zoology,* 61**,** 34-40.

HURST, R. J., LEONARD, M. L., WATTS, P. D., BECKERTON, P. & ØRITSLAND, N. A. (1982) Polar bear locomotion: body temperature and energetic cost. *Canadian Journal of Zoology,* 60**,** 40-44.

HUXLEY, A. F. (1974) Musclular contraction. *Journal of Physiology,* 243**,** 1-43.

JENSEN, T. F. & HOLM-JENSEN, I. (1980) Energetic cost of running in workers of 3 ant species, *Formica fusca*, *Formica rufa* and *Camponotus herculeanus* (Hymenoptera, Formicidae). *Journal of Comparative Physiology B,* 137**,** 151-156.

JETZ, W., THOMAS, G. H., JOY, J. B., HARTMANN, K. & MOOERS, A. O. (2012) The global diversity of birds in space and time. *Nature,* 491**,** 444-448.

JOHN-ALDER, H. B. & BENNETT, A. F. (1981) Thermal dependence of endurance and locomotory energetics in a lizard, *Dipsosaurus dorsalis*. *American Journal of Physiology,* 241**,** R342-R349.

JOHN-ALDER, H. B., GARLAND, T., JR & BENNETT, A. F. (1986) Locomotory capacities, oxygen consumption, and the cost of locomotion of the shingle-back lizard (*Trachydosaurus rugosus*). *Physiological Zoology,* 59**,** 523-531.

JOHN-ALDER, H. B., LOWE, C. H. & BENNETT, A. F. (1983) Thermal dependence of locomotory energetics and aerobic capacity of the gila monster *Heloderma suspectum*. *Journal of Comparative Physiology B,* 151**,** 119-126.

KABAT, A. P., PHILLIPS, R. A., CROXALL, J. P. & BUTLER, P. J. (2007) Differences in metabolic costs of terrestrial mobility in two closely related species of albatross. *Journal of Experimental Biology,* 210**,** 2851-2858.

KAMBHAMPATI, S. (1995) A phylogeny of cockroaches and related insects based on DNA sequence of mitochondrial ribosomal RNA genes. *Proceedings of the National Academy of Sciences of the United States of America,* 92**,** 2017-2020.

KRAM, R. (2012) Taylor's treadmill menagerie. *Journal of Experimental Biology,* 215**,** 2349-2350.

KRAM, R. & TAYLOR, C. R. (1990) Energetics of running: a new perspective. *Nature,* 346**,** 265-267.

LANGMAN, V. A., ROBERTS, T. J., BLACK, J., MALOIY, G. M. O., HEGLUND, N. C., WEBER, J.-M., KRAM, R. & TAYLOR, C. R. (1995) Moving cheaply: energetics of walking in the African elephant. *Journal of Experimental Biology,* 198**,** 629-632.

LANGMAN, V. A., ROWE, M. F., ROBERTS, T. J., LANGMAN, N. V. & TAYLOR, C. R. (2012) Minimum cost of transport in Asian elephants: do we really need a bigger elephant? *Journal of Experimental Biology,* 215**,** 1509-1514.

LAWLER, J. M., POWERS, S. K., HAMMEREN, J. & MARTIN, A. D. (1993) Oxygen cost of treadmill running in 24-month-old Fischer-344 rats. *Medicine and Science in Sports and Exercise,* 25**,** 1259-1264.

LEES, J. J., NUDDS, R. L., FOLKOW, L. P., STOKKAN, K.-A. & CODD, J. R. (2012) Understanding sex differences in the cost of terrestrial locomotion. *Proceedings of the Royal Society B: Biological Sciences,* 279**,** 826-832.

LIGHTON, J. R. B. (1985) Minimum cost of transport and ventilatory patterns in three African beetles. *Physiological Zoology,* 58**,** 390-399.

LIGHTON, J. R. B., BARTHOLOMEW, G. A. & FEENER, D. H. (1987) Energetics of locomotion and load carriage and a model of the energy cost of foraging in the leaf-cutting ant *Atta colombica* Guer. *Physiological Zoology,* 60**,** 524-537.

LIGHTON, J. R. B. & DUNCAN, F. D. (1995) Standard and exercise metabolism and the dynamics of gas exchange in the giant red velvet mite, *Dinothrombium magnificum*. *Journal of Insect Physiology,* 41**,** 877-884.

LIGHTON, J. R. B. & FEENER, D. H. (1989) A comparison of energetics and ventilation of desert ants during voluntary and forced locomotion. *Nature,* 342**,** 174-175.

LIGHTON, J. R. B. & GILLESPIE, R. G. (1989) The energetics of mimicry: the cost of pedestrian transport in a formicine ant and its mimic, a clubionid spider. *Physiological Entomology,* 14**,** 173-177.

LIGHTON, J. R. B. & HALSEY, L. G. (2011) Flow-through respirometry applied to chamber systems: Pros and cons, hints and tips. *Comparative Biochemistry and Physiology A,* 158**,** 265-275.

LIGHTON, J. R. B., WEIER, J. A. & FEENER, D. H. (1993) The energetics of locomotion and load carriage in the desert harvester ant *Pogonomyrmex rugosus*. *Journal of Experimental Biology,* 181**,** 49-61.

LIPP, A., WOLF, H. & LEHMANN, F.-O. (2005) Walking on inclines: energetics of locomotion in the ant *Camponotus*. *Journal of Experimental Biology,* 208**,** 707-719.

LUICK, B. R. & WHITE, R. G. (1986) Oxygen consumption for locomotion by caribou calves. *Journal of Wildlife Management,* 50**,** 148-152.

MAHONEY, S. A. (1980) Cost of locomotion and heat balance during rest and running from 0 to 55°C in a patas monkey. *Journal of Applied Physiology,* 49**,** 789-800.

MALOIY, G. M. O., RUGANGAZI, B. M. & ROWE, M. F. (2009) Energy expenditure during level locomotion in large desert ungulates: the one-humped camel and the domestic donkey. *Journal of Zoology,* 277**,** 248-255.

MARTINS, E. P. & HANSEN, T. F. (1997) Phylogenies and the comparative method: A general approach to incorporating phylogenetic information into the analysis of interspecific data. *American Naturalist,* 149**,** 646-667.

MISOF, B., LIU, S., MEUSEMANN, K., PETERS, R. S., DONATH, A., MAYER, C., FRANDSEN, P. B., WARE, J., FLOURI, T., BEUTEL, R. G., NIEHUIS, O., PETERSEN, M., IZQUIERDO-CARRASCO, F., WAPPLER, T., RUST, J., ABERER, A. J., ASPÖCK, U., ASPÖCK, H., BARTEL, D., BLANKE, A., BERGER, S., BÖHM, A., BUCKLEY, T. R., CALCOTT, B., CHEN, J., FRIEDRICH, F., FUKUI, M., FUJITA, M., GREVE, C., GROBE, P., GU, S., HUANG, Y., JERMIIN, L. S., KAWAHARA, A. Y., KROGMANN, L., KUBIAK, M., LANFEAR, R., LETSCH, H., LI, Y., LI, Z., LI, J., LU, H., MACHIDA, R., MASHIMO, Y., KAPLI, P., MCKENNA, D. D., MENG, G., NAKAGAKI, Y., NAVARRETE-HEREDIA, J. L., OTT, M., OU, Y., PASS, G., PODSIADLOWSKI, L., POHL, H., VON REUMONT, B. M., SCHÜTTE, K., SEKIYA, K., SHIMIZU, S., SLIPINSKI, A., STAMATAKIS, A., SONG, W., SU, X., SZUCSICH, N. U., TAN, M., TAN, X., TANG, M., TANG, J., TIMELTHALER, G., TOMIZUKA, S., TRAUTWEIN, M., TONG, X., UCHIFUNE, T., WALZL, M. G., WIEGMANN, B. M., WILBRANDT, J., WIPFLER, B., WONG, T. K. F., WU, Q., WU, G., XIE, Y., YANG, S., YANG, Q., YEATES, D. K., YOSHIZAWA, K., ZHANG, Q., ZHANG, R., ZHANG, W., ZHANG, Y., ZHAO, J., ZHOU, C., ZHOU, L., ZIESMANN, T., ZOU, S., LI, Y., XU, X., ZHANG, Y., YANG, H., WANG, J., WANG, J., KJER, K. M., et al. (2014) Phylogenomics resolves the timing and pattern of insect evolution. *Science,* 346**,** 763-767.

MOBERLY, W. R. (1968) The metabolic responses of the common iguana, *Iguana iguana*, to walking and diving. *Comparative Biochemistry and Physiology,* 27**,** 21-32.

NAKATSUKASA, M., OGIHARA, N., HAMADA, Y., GOTO, Y., YAMADA, M., HIRAKAWA, T. & HIRASAKI, E. (2004) Energetic costs of bipedal and quadrupedal walking in Japanese macaques. *American Journal of Physical Anthropology,* 124**,** 248-256.

NOLET, B. A., BUTLER, P. J., MASMAN, D. & WOAKES, A. J. (1992) Estimation of daily energy expenditure from heart rate and doubly labelled water in exercising geese. *Physiological Zoology,* 65**,** 1188-1216.

NUDDS, R. L., FOLKOW, L. P., LEES, J. J., TICKLE, P. G., STOKKAN, K.-A. & CODD, J. R. (2011) Evidence for energy savings from aerial running in the Svalbard rock ptarmigan (*Lagopus muta hyperborea*). *Proceedings of the Royal Society B: Biological Sciences,* 278**,** 2654-2661.

ORME, D., FRECKLETON, R. P., THOMAS, G. H., PETZOLDT, Y., FRITZ, S., ISAAC, N. & PEARSE, W. (2013) caper: Comparative Analyses of Phylogenetics and Evolution in R. R package version 0.5.2. ed.

ORON, U., CROMPTON, A. W. & TAYLOR, C. R. (1981) Energetic cost of locomotion in some 'primitive' mammals. *Physiological Zoology,* 54**,** 463-469.

PAGEL, M. (1999) Inferring the historical patterns of biological evolution. *Nature,* 401**,** 877-884.

PARADIS, E., CLAUDE, J. & STRIMMER, K. (2004) APE: Analyses of Phylogenetics and Evolution in R language. *Bioinformatics,* 20**,** 289-290.

PARKER, K. L., ROBBINS, C. T. & HANLEY, T. A. (1984) Energy expenditures for locomotion by mule deer (*Odocoileus hemionus*) and elk (*Cervus elaphus nelsoni*). *Journal of Wildlife Management,* 48**,** 474-488.

PARSONS, P. E. & TAYLOR, C. R. (1977) Energetics of brachiation versus walking: A comparison of a suspended and an inverted pendulum mechanism. *Physiological Zoology,* 50**,** 182-188.

PINSHOW, B., FEDAK, M. A., BATTLES, D. R. & SCHMIDT-NIELSEN, K. (1976) Energy expenditure for thermoregulation and locomotion in emperor penguins. *American Journal of Physiology,* 231**,** 903-912.

PINSHOW, B., FEDAK, M. A. & SCHMIDT-NIELSEN, K. (1977) Terrestrial locomotion in penguins: it costs more to waddle. *Science,* 195**,** 592-594.

PONTZER, H. (2007) Effective limb length and the scaling of locomotor cost in terrestrial animals. *Journal of Experimental Biology,* 210**,** 1752-1761.

PYRON, R. A., BURBRINK, F. T. & WIENS, J. J. (2013) A phylogeny and revised classification of Squamata, including 4161 species of lizards and snakes. *BMC Evolutionary Biology,* 13**,** 93.

PYRON, R. A. & WIENS, J. J. (2011) A large-scale phylogeny of Amphibia including over 2800 species, and a revised classification of extant frogs, salamanders, and caecilians. *Molecular Phylogenetics and Evolution,* 61**,** 543-583.

R CORE TEAM (2013) R: A Language and Environment for Statistical Computing. 3.0.0 ed. Vienna, Austria, R Foundation for Statistical Computing.

RALL, J. A. (1985) Energetic aspects of skeletal muscle contraction: implications of fiber types. *Exercise and Sport Sciences Reviews,* 13**,** 33-74.

REILLY, S. M., MCELROY, E. J. & BIKNEVICIUS, A. R. (2007) Posture, gait and the ecological relevance of locomotor costs and energy-saving mechanisms in tetrapods. *Zoology,* 110**,** 271-289.

REZENDE, E. L. & DINIZ-FILHO, J. A. F. (2012) Phylogenetic analyses: comparing species to infer adaptations and physiological mechanisms. *Comprehensive Physiology,* 2**,** 639-674.

REZENDE, E. L., GOMES, F. R., CHAPPELL, M. A. & GARLAND, T., JR (2009) Running behavior and its energy cost in mice selectively bred for high voluntary locomotor activity. *Physiological and Biochemical Zoology,* 82**,** 662-679.

REZENDE, E. L., KELLY, S. A., GOMES, F. R., CHAPPELL, M. A. & GARLAND, T., JR (2006) Effects of size, sex, and voluntary running speeds on costs of locomotion in lines of laboratory mice selectively bred for high wheel-running activity. *Physiological and Biochemical Zoology,* 79**,** 83-99.

ROBERTS, T., KRAM, R., WEYAND, P. & TAYLOR, C. R. (1998a) Energetics of bipedal running. I. Metabolic cost of generating force. *Journal of Experimental Biology,* 201**,** 2745-2751.

ROBERTS, T. J., CHEN, M. S. & TAYLOR, C. R. (1998b) Energetics of bipedal running. II. Limb design and running mechanics. *Journal of Experimental Biology,* 201**,** 2753-2762.

ROBERTS, T. J., KRAM, R., WEYAND, P. G. & TAYLOR, C. R. (1998c) Energetics of bipedal running I. Metabolic cost of generating force. *Journal of Experimental Biology,* 201**,** 2745-2751.

ROSE, K. A., TICKLE, P. G., LEES, J. J., STOKKAN, K.-A. & CODD, J. R. (2014) Neither season nor sex affects the cost of terrestrial locomotion in a circumpolar diving duck: the common eider (*Somateria mollissima*). *Polar Biology,* 37**,** 879-889.

SCANTLEBURY, D. M., MILLS, M. G. L., WILSON, R. P., WILSON, J. W., MILLS, M. E. J., DURANT, S. M., BENNETT, N. C., BRADFORD, P., MARKS, N. J. & SPEAKMAN, J. R. (2014) Flexible energetics of cheetah hunting strategies provide resistance against kleptoparasitism. *Science,* 346**,** 79-81.

SCHILMAN, P. E. & ROCES, F. (2005) Energetics of locomotion and load carriage in the nectar feeding ant, *Camponotus rufipes*. *Physiological Entomology,* 30**,** 332-337.

SCHMIDT-NIELSEN, K. (1972a) Locomotion: energy cost of swimming, flying and running. *Science,* 172**,** 222-228.

SCHMIDT-NIELSEN, K. (1972b) Locomotion: Energy cost of swimming, flying, and running. *Science,* 177**,** 222-228.

SCHMIDT-NIELSEN, K. (1984) *Scaling: Why is animal size so important?,* Cambridge, Cambridge University Press.

SCHMITZ, A. (2005) Spiders on a treadmill: influence of running activity on metabolic rates in *Pardosa lugubris* (Araneae, Lycosidae) and *Marpissa muscosa* (Araneae, Salticidae). *Journal of Experimental Biology,* 208**,** 1401-1411.

SEEBACHER, F., WHITE, C. R. & FRANKLIN, C. E. (2015) Physiological plasticity increases resilience of ectothermic animals to climate change. *Nature Climate Change,* 5**,** 61-66.

SEEHERMAN, H. J., TAYLOR, C. R., MALOIY, G. M. O. & ARMSTRONG, R. B. (1981) Design of the mammalian respiratory system. II. Measuring maximum aerobic capacity. *Respiration Physiology,* 44**,** 11-23.

SHILLINGTON, C. & PETERSON, C. C. (2002) Energy metabolism of male and female tarantulas (*Aphonopelma anax*) during locomotion. *Journal of Experimental Biology,* 205**,** 2909-2914.

SPEAKMAN, J. R. & SELMAN, C. (2003) Physical activity and resting metabolic rate. *Proceedings of the Nutrition Society,* 62**,** 621-634.

SYMONDS, M. R. E. & MOUSSALLI, A. (2011) A brief guide to model selection, multimodel inference and model averaging in behavioural ecology using Akaike’s information criterion. *Behavioral Ecology and Sociobiology,* 65**,** 13-21.

TAYLOR, C., HEGLUND, N. & MALOIY, G. (1982a) Energetics and mechanics of terrestrial locomotion. I. Metabolic energy consumption as a function of speed and body size in birds and mammals. *J Exp Biol,* 97**,** 1-21.

TAYLOR, C., SCHMIDT-NIELSEN, K. & RAAB, J. (1970a) Scaling of energetic cost of running to body size in mammals. *American Journal of Physiology,* 219**,** 1104-1107.

TAYLOR, C. R., CALDWELL, S. L. & ROWNTREE, V. J. (1972) Running up and down hills: Some consequences of size. *Science,* 178**,** 1096-1097.

TAYLOR, C. R., DMI'EL, R., FEDAK, M. A. & SCHMIDT-NIELSEN, K. (1971a) Energetic cost of running and heat balance in a large bird, the rhea. *American Journal of Physiology,* 221**,** 597-601.

TAYLOR, C. R., HEGLUND, N. C. & MALOIY, G. M. O. (1982b) Energetics and mechanics of terrestrial locomotion: I. Metabolic energy consumption as a function of speed and body size in birds and mammals. *Journal of Experimental Biology,* 97**,** 1-22.

TAYLOR, C. R. & ROWNTREE, V. J. (1973) Running on two or on four legs: which consumes more energy? *Science,* 179**,** 186-187.

TAYLOR, C. R., SCHMIDT-NIELSEN, K., DMI'EL, R. & FEDAK, M. A. (1971b) Effect of hyperthermia on heat balance during running in the African hunting dog. *American Journal of Physiology,* 220**,** 823-827.

TAYLOR, C. R., SCHMIDT-NIELSEN, K. & RAAB, J. L. (1970b) Scaling of energetic cost of running to body size in mammals. *American Journal of Physiology,* 219**,** 1104-1107.

TAYLOR, C. R., SHKOLNIK, A., DMI'EL, R., BAHARAV, D. & BORUT, A. (1974) Running in cheetahs, gazelles, and goats: energy cost and limb configuration. *American Journal of Physiology,* 227**,** 848-850.

THOMPSON, S. D., MACMILLEN, R. E., BURKE, E. M. & TAYLOR, C. R. (1980) The energetic cost of bipedal hopping in small mammals. *Nature,* 287**,** 223-224.

VAILLANCOURT, E., PRUD’HOMME, S., HAMAN, F., GUGLIELMO, C. G. & WEBER, J.-M. (2005) Energetics of a long-distance migrant shorebird (*Philomachus pugnax*) during cold exposure and running. *Journal of Experimental Biology,* 208**,** 317-325.

WALTON, B. M., PETERSON, C. C. & BENNETT, A. F. (1994) Is walking costly for anurans? The energetic cost of walking in the northern toad *Bufo boreas halophilus*. *Journal of Experimental Biology,* 197**,** 165-78.

WALTON, M. & ANDERSON, B. D. (1988) The aerobic cost of saltatory locomotion in the fowler's toad (*Bufo woodhousei fowleri*). *Journal of Experimental Biology,* 136**,** 273-288.

WARD, P. S. (2007) Phylogeny, classification, and species-level taxonomy of ants (Hymenoptera: Formicidae). *Zootaxa,* 1668**,** 549-563.

WEIER, J. A., FEENER, D. H., JR. & LIGHTON, J. R. B. (1995) Inter-individual variation in energy cost of running and loading in the seed-harvester ant, *Pogonomyrmex maricopa*. *Journal of Insect Physiology,* 41**,** 321-327.

WHITE, C. R., GRÉMILLET, D., GREEN, J. A., MARTIN, G. R. & BUTLER, P. J. (2011) Metabolic rate throughout the annual cycle reveals the demands of an Arctic existence in Great Cormorants. *Ecology,* 92**,** 475-486.

WHITE, C. R. & KEARNEY, M. R. (2014) Metabolic scaling in animals: methods, empirical results, and theoretical explanations. *Comprehensive Physiology,* 4**,** 231-256.

WHITE, C. R., MARTIN, G. R. & BUTLER, P. J. (2008a) Pedestrian locomotion energetics and gait characteristics of a diving bird, the great cormorant, *Phalacrocorax carbo*. *Journal of Comparative Physiology B,* 178**,** 745–754.

WHITE, C. R., TERBLANCHE, J. S., KABAT, A. P., BLACKBURN, T. M., CHOWN, S. L. & BUTLER, P. J. (2008b) Allometric scaling of maximum metabolic rate: the influence of temperature. *Functional Ecology,* 22**,** 616-623.

WICKLER, S. J., HOYT, D. F., COGGER, E. A. & MYERS, G. (2003) The energetics of the trot–gallop transition. *Journal of Experimental Biology,* 206**,** 1557-1564.

WILLIAMS, T. M. (1983) Locomotion in the North American mink, a semi-aquatic mammal II. The effect of an elongate body on running energetics and gait patterns. *Journal of Experimental Biology,* 105**,** 283-295.

WILLIAMS, T. M., BEN-DAVID, M., NOREN, S., RUTISHAUSER, M., MCDONALD, K. & HEYWARD, W. (2002) Running energetics of the North American river otter: do short legs necessarily reduce efficiency on land? *Comparative Biochemistry and Physiology A,* 133**,** 203-212.

WILLIAMS, T. M., WOLFE, L., DAVIS, T., KENDALL, T., RICHTER, B., WANG, Y., BRYCE, C., ELKAIM, G. H. & WILMERS, C. C. (2014) Instantaneous energetics of puma kills reveal advantage of felid sneak attacks. *Science,* 346**,** 81-85.

WUNDER, B. A. (1970) Energetics of running activity in Merriam's chipmunk, *Eutamias merriami*. *Comparative Biochemistry and Physiology,* 33**,** 821-836.

WUNDER, B. A. & MORRISON, P. R. (1974) Red squirrel metabolism during incline running. *Comparative Biochemistry and Physiology Part A: Physiology,* 48**,** 153-161.

YOUSEF, M. K., ROBERTSON, W. D., DILL, D. B. & JOHNSON, H. D. (1970) Energy expenditure of running kangaroo rats *Dipodomys merriami*. *Comparative Biochemistry and Physiology,* 36**,** 387-393.

YOUSEF, M. K., ROBERTSON, W. D., DILL, D. B. & JOHNSON, H. D. (1973) Energetic cost of running in the antelope ground squirrel *Ammospermophilus leucurus*. *Physiological Zoology,* 46**,** 139-147.

ZANI, P. A. & KRAM, R. (2008) Low metabolic cost of locomotion in ornate box turtles, *Terrapene ornata*. *Journal of Experimental Biology,* 211**,** 3671-3676.

ZEFFER, A., JOHANSSON, L. C. & MARMEBRO, Å. (2003) Functional correlation between habitat use and leg morphology in birds (Aves). *Biological Journal of the Linnean Society,* 79**,** 461-484.
